# DNA Barcoding Silver Butter Catfish (*Schilbe intermedius*) Reveals Patterns of Mitochondrial Genetic Diversity Across African River Systems

**DOI:** 10.1038/s41598-020-63837-4

**Published:** 2020-04-27

**Authors:** Lotanna M. Nneji, Adeniyi C. Adeola, Moshood K. Mustapha, Segun O. Oladipo, Chabi A. M. S. Djagoun, Ifeanyi C. Nneji, Babatunde E. Adedeji, Omotoso Olatunde, Adeola O. Ayoola, Agboola O. Okeyoyin, Odion O. Ikhimiukor, Galadima F. Useni, Oluyinka A. Iyiola, Emmanuel O. Faturoti, Moise M. Matouke, Wanze K. Ndifor, Yun-yu Wang, Jing Chen, Wen-Zhi Wang, Jolly B. Kachi, Obih A. Ugwumba, Adiaha A. A. Ugwumba, Christopher D. Nwani

**Affiliations:** 1 State Key Laboratory of Genetic Resources and Evolution, Kunming Institute of Zoology, Chinese Academy of Sciences, Kunming, 650223 China; 2 Sino-Africa Joint Research Centre, Chinese Academy of Sciences, Kunming, China; 3 Department of Zoology, Faculty of Life Sciences, University of Ilorin, Ilorin, Kwara State Nigeria; 4 Department of Biosciences and Biotechnology, College of Pure and Applied Sciences, Kwara State University, Malete, Kwara State Nigeria; 5 Laboratory of Applied Ecology, Faculty of Agronomic Sciences, University of Abomey-Calavi, Abomey-Calavi, Benin; 6 Department of Biological Science, Faculty of Sciences, University of Abuja, Abuja, Nigeria; 7 Department of Zoology, Faculty of Science, University of Ibadan, Ibadan, Oyo State Nigeria; 8 National Park Service Headquarter, Federal Capital Territory, Abuja, Nigeria; 9 Department of Microbiology, Faculty of Science, University of Ibadan, Ibadan, Oyo State Nigeria; 10 Taraba State Polytechnic, Suntai, Taraba State Nigeria; 11 Department of Aquaculture and Fisheries Management, Faculty of Agriculture, University of Ibadan, Ibadan, Oyo State Nigeria; 12 Department of Zoology, Faculty of Science, University of Douala, Douala, Cameroon; 13 Department of Zoology, Faculty of Science, University of Dschang, Dschang, Cameroon; 14 Wild Forensic Center, Kunming, China; 15 Department of Biological Science, Faculty of Sciences, Federal University Lokoja, Lokoja, Nigeria; 16 Department of Zoology and Environmental Biology, Faculty of Biological Sciences, University of Nigeria, Nsukka, Nigeria

**Keywords:** Freshwater ecology, Evolutionary biology

## Abstract

The silver butter catfish (*Schilbe intermedius*) is widely distributed across African river systems. To date, information on its mitochondrial genetic diversity, population structure, and historical demography are not well-established. Herein, we combined newly generated mitochondrial cytochrome c oxidase (*COI*) subunit I gene sequences with previously published *COI* sequences in the global databases to reconstruct its phylogeography, population genetic structure, and historical demography. Results from the mtDNA phylogeography and species delimitation tests (Cluster algorithm – Species Identifier, Automatic Barcode Gap Discovery and Poison Tree Process model) revealed that *S. intermedius* comprises at least seven geographically defined matrilines. Although the overall haplotype diversity of *S. intermedius* was high (h = 0.90), results showed that East (Kenya) and West (Nigeria) African populations had low levels of haplotype diversity (h = ~0.40). In addition, population genetic polymorphism and historical demographics showed that *S. intermedius* populations in both East and West Africa underwent severe contractions as a result of biogeographic influences. The patterns of genetic diversity and population structure were consistent with adaptive responses to historical biogeographic factors and contemporary environmental variations across African river systems. This is suggestive of the influence of historical biogeographic factors and climatic conditions on population divergence of *S. intermedius* across African river systems. Given our discovery of previously underappreciated diversity within *S. intermedius*, we recommend that this species be considered for increased conservation and management.

## Introduction

Freshwater fishes have been reported to show remarkable morphological variations across river systems. This could be attributed to morphometric characteristic changes associated with ontogenetic development and/or species’ local adaptation to its environment^1^. As such, the use of morphometric and meristic characters in taxonomy, particularly for species with extremely similar external features and high levels of phenotypic plasticity, could hinder the discovery and identification of cryptic intraspecific diversity^1–5^. While morphology-based taxonomy might possibly overlook such diversity, DNA barcoding, involving mitochondrial cytochrome c oxidase subunit I (*COI*), has been recommended as a complementary taxonomic tool for species identification and unravelling cryptic diversity^6^. The *COI* barcoding has proven successful in the identification of fish species^1–5,7^ and the discovery of cryptic diversities^2,8–11^. Several examples of deep *COI* sequence divergences within fish species have been attributed to cryptic speciation^2,3,8–11^.

More than 3360 fish species have been described in African river systems^12,13^. These organisms are important and often underappreciated component of African biodiversity. Additionally, these fishes are significant economic and food resources for residents of African river watersheds^2^. Given their importance to humans, the ability to accurately identify species and other evolutionary distinct units are prerequisites for sustainable management of fish genetic resources and improving our knowledge of the diversity of African riverine fish diversity^2–5^.

*Schilbe intermedius*, commonly known as silver catfish or butterfish, is a potamodromous fish that migrates between different freshwater bodies^11^. It is widely distributed and abundant throughout sub-Saharan African river systems^14^. Traditionally, external morphological characteristics have been used to differentiate species within the family Schilbeidae^11^. For example, the absence of an adipose fin was used to differentiate southern African silver catfish as belonging to the genus *Schilbe* and the members of the similar-looking genus *Eutropius* with an adipose fin^11,15^. Nonetheless, the absence of an adipose fin for species within the genus *Schilbe* has been found unreliable as a key morphological feature for the identification of *S. intermedius*^11^. For example, while populations of *S. intermedius* from southern African river systems (Namibia and Botswana) lack adipose fins, adipose fins are present on other southern African populations. Additionally, rudimentary adipose fins were also observed in populations from West (Nigeria) and East (Kenya) African river systems^11,15,16^.

Several molecular phylogenetic studies based on DNA barcode data have explored the mitochondrial diversity of *S. intermedius*^2,3,11^, though the results of these studies were equivocal. For instance, while Van der Bank *et al*.^11^ recovered three distinct matrilines across African river systems (South Africa, East Africa, and West Africa), Iyiola *et al*.^2^ revealed the presence of four geographically structured matrilines with two restricted to West Africa (Nigeria) and the other two to East and Central Africa. Nonetheless, these studies strongly suggest that the genetic diversity of *S. intermedius* remain under-estimated. In particular, a lack of extensive taxon sampling particularly from Nigeria^2,11^ limits our understanding of the population-level diversity within this species.

In this study, we expand on previous efforts directed towards the extensive phylogeographic analysis of *S. intermedius* based on *COI* sequence data. Combining our newly generated sequences with the available sequences in global databases, we reconstructed the mtDNA phylogeography of *S. intermedius* with the aim of investigating its species boundaries as well as to unravel its patterns of genetic diversity, population structure and historical demography across African river systems. This study improves knowledge on the mitochondrial *COI* genetic diversity and population structuring of *S. intermedius* across African river systems. Further, the DNA barcode records generated in this study will be available to researchers and biodiversity managers for monitoring and mapping out effective conservation measures for African fisheries.

## Results

### Sequence information

**Figure 1:**
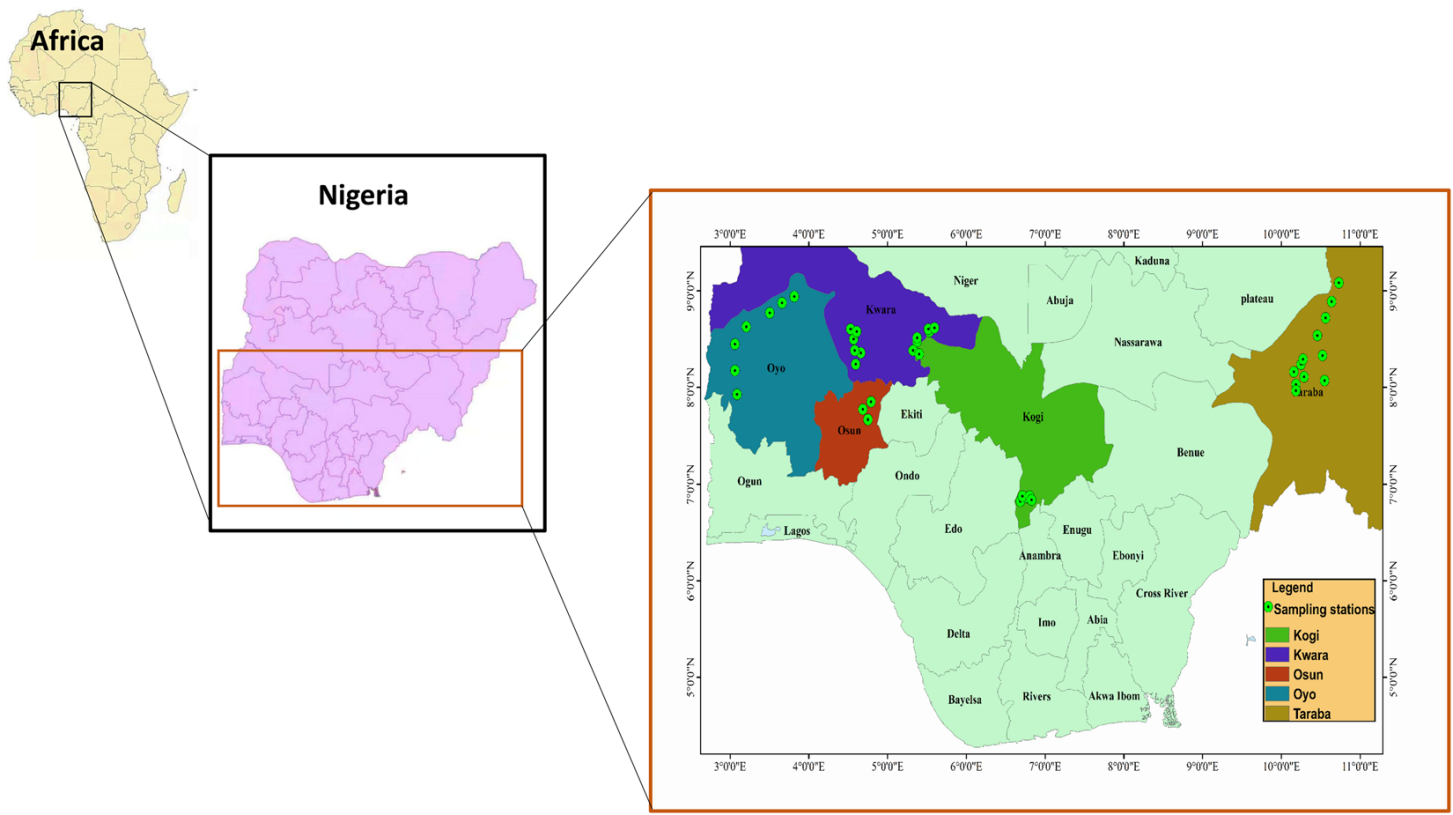
Map of collection sites for the newly sampled *Schilbe intermedius* in Nigeria.

We amplified *COI* sequences of 118 specimens of *S. intermedius* collected from different river bodies in Nigeria (Fig. 1). By combining our sequences with the previously published *COI* sequences of *S. intermedius* in global databases (Supplementary Table S1), we aligned a total of 648 base pairs of *COI* sequence data. Among these sites, 150 were variable and 119 were potentially parsimony informative. The average nucleotide frequencies were A = 25.90%, T = 27.60%, C = 29.30%, and G = 17.30%. The *COI* gene for *S. intermedius* consisted of 31 unique haplotypes with overall haplotype diversity (h) = 0.90, nucleotide diversity (π) = 0.05 and mean number of pairwise differences (k) = 21.705. Newly generated sequences were deposited in GenBank under Accession Numbers MN509590 – MN509707 (Supplementary Table S1).

### Phylogenetic reconstruction and species delimitation test

**Figure 2:**
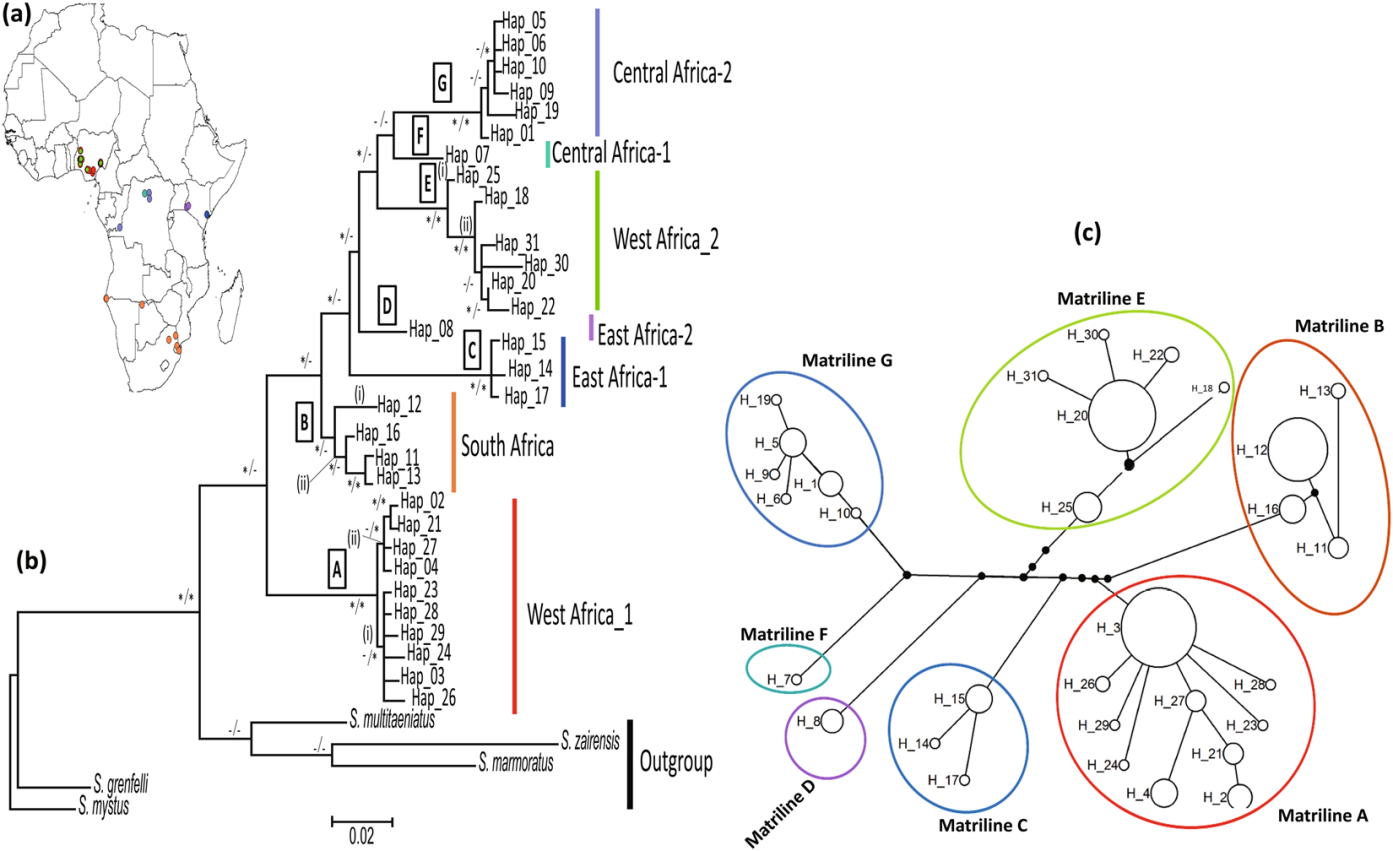
(**a**) Map showing the geographic distribution of matrilines of *Schilbe intermedius* in sub-Saharan Africa; (**b**) Phylogenetic tree inferred from ML analysis of *S. intermedius* based on mtDNA *COI* data set. Support values at each node are Bayesian posterior probability (left) and bootstrap values from ML (right). Asterisk (*) indicates full support (Bayesian posterior probability for BI ≥ 0.95, bootstrap proportions for ML ≥ 70%) in both analyses and hyphen (−) indicates moderate to weak support (Bayesian posterior probability for BI ≤ 0.95, bootstrap proportions for ML ≤ 70%). Letters A–G above the branches indicate matriline, while Roman numeral (i – ii) indicate sub-matriline; (**c**) Median-joining network of cytochrome c oxidase subunit I haplotypes of *S. intermedius* from the African river systems. Node sizes are proportional to total haplotype frequencies. The numbers on the internodes indicate mutation steps.

The BI and ML trees recovered similar topologies comprising seven matrilines ranging from moderate to strong support (Fig. 2a-b). Matriline A (West Africa-1) comprised individuals from Nigeria and further divided into two sub-matrilines (A (i–ii)). Matriline B (Southern Africa), included two sub-matrilines: B (i) restricted to South Africa and sub-matriline B (ii) consisting of populations from Botswana and Namibia. Matrilines C (East Africa-1) and D (East Africa-2) comprised populations from Kenya. Matriline E (West Africa-2) contained two sub-matrilines that included individuals from Nigeria. Matriline F (Central Africa-1) was restricted to the upper Congo River in the Democratic Republic of Congo (DRC), Central Africa while matriline G (Central Africa-2) consisted of individuals from the middle Congo River, Itimbiri River and Kinshaha in DRC. The relationships between the 31 unique haplotypes in *S. intermedius* populations are shown in the median-joining haplotype network (Fig. 2c). The similarity of the branching patterns of the median-joining haplotype network with the phylogenetic tree supports the presence of at least seven matrilines of *S. intermedius* across African river systems.

The number of species units identified by ‘clusters’ in SpeciesIdentifier (approach 1) was the same as the threshold values increased from 1.0% to 3.0% (Supplementary Table S2). Using the distance threshold, seven clusters were recovered which corroborated with the matrilines identified by BI and ML phylogenetic analyses (Supplementary Table S2). Further, the ABGD test (approach 2) supported the presence of multiple cryptic species-units within *S. intermedius* (Fig. S1). Similarly, our ABGD result was congruent with the phylogeny constructed using BI and ML approaches. Partition with a prior maximal distance of P = 0.0359 for both K2P and JC69 delimited the dataset into seven species units (Supplementary Fig. S1; Supplementary Table S3). The PTP with the best-fit ML search (approach 3) recovered additional lineages (10 lineages in total; Supplementary Table S4) when compared to the SpeciesIdentifier and ABGD approaches. However, most of the additional lineages were sub-lineages previously identified in our ML and BI analyses (Supplementary Table S4). As the PTP model tends to overestimate the number of potential species unit, we took a conservative approach (approach 1) and ABGD results, and results of the clustering/partitioning analyses to designate species units.

### Population genetic analyses and historical demography

**Figure 3:**
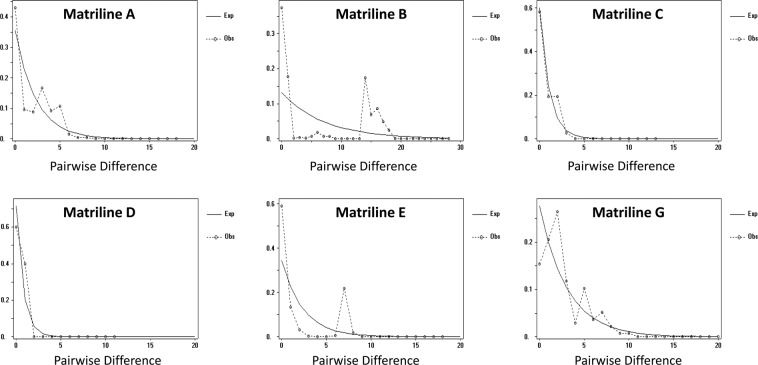
Population demographics for matrilines of *Schilbe intermedius* based on mtDNA *COI* dataset. Matriline F was excluded as it was represented by one unique haplotype.

Matriline A (West Africa-1) contained the highest number of haplotype (H = 13) while the smallest number (H = 2) was recorded in matriline D (East Africa-2; Table 1). The highest haplotype diversity (h = 0.846) was recorded in matriline G (Central Africa-2) and the lowest in matrilines C (East Africa-1), D (East Africa-2) and E (West Africa-2). On the other hand, the nucleotide diversity (π) for all matrilines was generally low ranging from 0.062% to 1.03% (Table 1). The mismatch distribution of frequencies of pairwise nucleotide differences (Fig. 3) showed a multimodal curve for matrilines A (West Africa-1) and G (Central Africa-2); however, the bimodal curve observed for matrilines B (South Africa) and E (West Africa-2) indicates the existence of two or more haplogroups within these matrilines (c.f. Figure 3). The unimodal distribution observed for matrilines C (East Africa-1) and D (East Africa-2) suggested population expansion (Fig. 3). In all cases, mismatch distribution test statistics (SSD and Raggedness Index) were relatively small and not significant (Table 2). Additionally, the neutrality tests yielded significant negative values for most matrilines (Table 2).

**Table 1:** Population genetic polymorphism of *Schilbe intermedius* based on mtDNA *COI* dataset.

Nucleotide Polymorphism	Matriline					
	A	B	C	D	E	G
Sample size	82	48	9	5	58	17
Number of polymorphic sites	16	22	3	1	11	16
Number of haplotype	13	8	3	2	7	9
Haplotype (gene) diversity	0.571	0.62	0.417	0.400	0.410	0.846
Nucleotide diversity (%)	0.333	1.03	0.103	0.062	0.35	0.579
Average number of pairwise nucleotide differences	1.816	6.577	0.66	0.400	1.91	2.61

Note: Matriline F was excluded as it was represented by single unique haplotype.

**Table 2:** Population demography statistics of *Schilbe intermedius* based on mtDNA *COI* dataset.

Matrilines	A	B	C	D	E	G
Fu & Li’s D	−2.640	1.039	−1.682	−0.817	−0.346	−2.719
*P*	<0.05	>0.10	>0.10	>0.10	>0.10	<0.05
Fu & Li’s F	−2.592	1.241	−1.820	−0.772	−0.491	−2.819
*P*	<0.05	>0.10	>0.10	>0.10	>0.10	<0.05
Fu’s Fs	−3.94	5.288	−1.513	0.090	0.382	−2.817
*P*	0.33	>0.10	>0.10	>0.10	>0.10	0.10
Tajima’s D	−1.358	1.06	−0.380	−0.817	−0.553	−1.732
*P*	>0.10	>0.10	>0.10	>0.10	>0.10	0.10 > P > 0.05
SSD	0.073	0.135	0.250	NA	0.109	0.05
P_*SSD*_	0.22	0.09	0.00	NA	0.100	0.22
Raggedness Index	0.237	0.397	0.180	NA	0.447	0.179
P_*RAG*_	0.190	0.450	1.00	NA	0.370	0.21

Note: - NA; matrilines for which the variance of the mismatch distribution was too small and, SSD and Raggedness Index were not estimated. Population demographics were not estimated for Matriline F as it was represented by one unique haplotype.

## Discussion

Morphology-based identification within the Family Schilbeidae, particularly for that of *S. intermedius*, has been problematic. Our study, similar to Van Der Bank *et al*.^11^, confirms that the efficacy of the DNA barcoding approach for the identification of *S. intermedius*. Our study thus gives credence on the effectiveness of integrative taxonomy in the identification and diversity studies of African freshwater fishes. In part, our study provides reference DNA barcode data that can be used in subsequent ecological, fisheries, food, forensic and other types of studies.

The results of the mtDNA phylogeography, species delimitation (clustering analyses and ABGD) and population genetic analyses showed that *S. intermedius* consists of several geographically defined matrilines. The mtDNA phylogeography, consistent with the clustering analyses, further confirmed the presence of at least seven matrilines of *S. intermedius* in the African river systems. In addition, both BI and ML analyses recovered populations from matriline A (West Africa - Nigeria) as a sister-group to other matrilines from African river systems. Although our mtDNA genealogical tree was consistent with earlier studies^2,11^, our study adds new matrilines and sub-matrilines to previously identified matrilines. Similar to the previous studies^2,11^, we also observed strong biogeographic signals and population divergence within *S. intermedius*. This study, therefore, supports evidence of population divergence within *S. intermedius* across African river systems. Although we cannot rule out the possibility of an ancient biogeographic connection between *S. intermedius* distributed across African river systems, extensive sampling, and the use of multiple molecular markers would aid in testing this hypothesis.

Due to the limitation arising from the use of single locus in biogeographic studies, our analysis cannot test the hypothesis that environmental changes and geographic isolation are contributors to the diversification of *S. intermedius* in Africa. However, our phylogeographic study reveals the presence of two geographically restricted matrilines of *S. intermedius* in Nigeria, Kenya, and DRC. In concordance with previous studies^11,17^, this could indicate genetic adaptation of these populations in response to local selective challenges and environmental pressures. Although the paucity of genetic studies on African freshwater fishes hindered comparison, we note that previous studies^18,19^ have exemplified the roles of local adaptation to environmental changes on the population genetic structure of African fauna. Further studies are therefore needed to investigate the evolutionary mechanisms or processes governing the diversification of *S. intermedius* in these regions, as well as to estimate the divergence events that could have accounted for the prolonged period of isolation of the matrilines.

The population genetic structure analysis revealed an overall high genetic diversity for *S. intermedius*. However, results obtained showed that populations from East (matrilines C and D) and West (matriline E) Africa had low haplotype diversity. This finding is similar to a previous study that also reported low genetic diversity in an East African catfish, *Bagrus docmak*^18^. Other studies have associated low genetic diversity in fishes with founder events and population bottlenecks that occurred due to the recent introduction of species into the environment^20–22^. Thus, the patterns of genetic diversity observed in matrilines C, D, and E, are likely the results of bottlenecks or founder effects experienced by the progenitors of these matrilines. Additionally, it is known that, due to their potamodromous migrations, populations of *S. intermedius* throughout African river systems were influenced by the climatic oscillations during the Pleistocene Era^11,23^. These climatic events could have isolated the progenitors of these matrilines, accounting for the low genetic diversity we observed in this species.

Understanding biogeography using molecular data is important for the interpretation of the distribution patterns of geographically distant populations. High haplotype diversity coupled with low nucleotide diversity as was observed in *S. intermedius* populations, is a pattern consistent with other catfish species such as Chinese *Leiocassis longirostris*^24^ and East African *B. docmak*^18^. Our study, therefore, provides evidence that historical biogeographic factors and contemporary environmental variations across sub-Saharan Africa accounted for the population divergence and geographic structuring within *S. intermedius*.

The main limitations of our study lie on the use of single locus (mitochondrial DNA) in inferring phylogeny. This is because mitochondrial DNA is maternally inherited and it reveals only a small part of the evolutionary history of a species^25,26^. As previously reported, Ballard & Whitlock^27^ have argued that mtDNA evolution is non-neutral which raises concerns on its utility as a sole genetic marker for inferring evolutionary history. On the other hand, direct (selection on mtDNA) and indirect (selection arising from disequilibrium with other maternally transmitted genes) selection are sufficiently common when using mtDNA gene markers^28^. Thus, these limitations could affect the inferences that could be drawn from the phylogeographic studies that lies solely on mtDNA gene markers. Thus, we recommend the use of both multiple markers that would include mtDNA and nuclear genes for more detailed molecular phylogenetic studies and investigation of patterns of gene flow in *S. intermedius*.

In conclusion, our study shows that DNA barcoding is an effective complementary tool to morphology in the identification and diversity study of *S. intermedius*. Incorporation of newly acquired *COI* sequences with existing molecular data in the global databases allowed investigation of the genetic diversity, population structure and historical demographics of *S. intermedius* across African river systems. The results obtained proved that the DNA barcode approach is effective in identifying genetic clusters as well as revealing hidden mitochondrial diversity. Our study benefits from having wider coverage and inclusion of more DNA barcode data as compared to other studies, and, thus, expands our understanding on the patterns of mitochondrial diversity and population structure of *S. intermedius*. From a conservation standpoint, the best management strategy for *S. intermedius* would be to consider each matriline independently when devising plans for the preservation of this species. For example, Nigerian matrilines of *S. intermedius* are declining due to current rate of overfishing and exploitation^29,30^, and specific management policies and plans are needed for their conservation. Further studies, using a large number of gene loci including both mtDNA and nuclear genes, are required to ascertain (1) the evolutionary mechanism (s) driving the diversification of *S. intermedius* in Nigeria, Kenya, and DRC, (2) the timing of divergence events, and (3) evidence of nuclear gene flow upon secondary contact.

## Materials and Methods

### Ethical Statement

*Schilbe intermedius* is not protected under any legislation and not considered threatened or endangered. Samples from Nigeria were collected from non-protected areas for which permissions were not required as the sampling locations do not fall under the Nigerian Wildlife Protection Act.

### Sample collection

With the help of local fishermen and field assistants, 118 individuals of *S. intermedius* were collected from different freshwater bodies in Nigeria (Table S1) between July to December 2018. Individuals were collected using gill nets, hook and line, and/or cast net, and transported on ice to the Zoology Laboratory of the Department of Bioscience and Biotechnology, Kwara State University (KWASU), Malete, Nigeria. The preliminary species identification was in accordance with the taxonomic guidelines^2,31,32^. Additional species identification and verification were carried out by two trained taxonomists at the Department of Zoology, University of Ilorin, Nigeria. Tissue (tail fin) samples were collected and preserved in 95% ethanol and subsequently stored under −80 °C at the State Key Laboratory of Genetic Resources and Evolution, Kunming Institute of Zoology, Chinese Academy of Sciences, China. Vouchers were fixed with 4% formalin and kept in 70% ethanol for long-term storage at the Zoology Laboratory of the Department of Bioscience and Biotechnology, KWASU, Nigeria.

### DNA extraction, PCR amplification, and sequencing

Total genomic DNA was extracted from the ethanol-preserved tissues following the standard phenol-chloroform extraction procedure after digestion with proteinase K^33^. The *COI* gene fragment of the newly acquired specimens was amplified with primers^7^ in a volume reaction of 25 µl: 1.5 µl of genomic working DNA, 18.5 µl of PCR water, 2.5 µl of PCR buffer, 2 µl of dNTP, 1 µl of each of the forward and reverse primers (10 pm/µl) and 0.30 µl of rTaq polymerase. The PCR cycle profiles were as follow: 5 minutes initial denaturation at 94 °C, followed by 35 cycles of 1 minute at 94 °C, annealing for 45 seconds at 55 °C, an extension for 1 minute at 72 °C; final extension for 10 minutes at 72 °C. Purified PCR products were directly sequenced in both forward and reverse directions with an automated DNA sequencer (ABI 3730).

### DNA sequence alignment and dataset assembly

To confirm the identity of the amplified sequences, sequences were submitted to BLAST searches in National Center for Biotechnology Information- NCBI (https://blast.ncbi.nlm.nih.gov/Blast.cgi). Thereafter, 101 *COI* sequences of *S. intermedius* from West, South, East and Central Africa were downloaded from the NCBI (http://www.ncbi.nlm.nih.gov) and Barcode of Life Database (http://www.boldsystems.org/index.php/TaxBrowser_Home) (Table S1). Further, *COI* sequences of five closely related species (*Schilbe mystus*, *Schilbe multitaeniatus*, *Schilbe grenfelli, Schilbe marmoratus*, and *Schilbe zairensis*) were downloaded as outgroup taxa (Appendix 1). A total of 219 *COI* nucleotide sequences of *S. intermedius* and five outgroup taxa were aligned in MEGA 7.0^34^ using ClustalW^35^ with default parameters. The aligned sequences were translated into amino acids using the vertebrate mitochondrial code and no premature stop codons were observed, suggesting that the open reading frame was maintained in the protein-coding loci.

### Phylogenetic reconstruction and species delimitation tests

For the phylogenetic reconstruction, the sequence dataset was collapsed into 31 unique *COI* haplotypes of *S. intermedius* using DnaSP 5.10^36^. MtDNA phylogeny was reconstructed using Bayesian Inference (BI) and Maximum Likelihood (ML) approaches. The best partition strategy and nucleotide substitution model for the BI were selected using the Akaike information criterion (AIC) as implemented in PartitionFinder 1.0.1^37^. Following analysis using Partition Finder, the mtDNA *COI* sequence dataset was partitioned into codon 1, 2 and 3, and the best-fitting models were selected for each of the partitioned data. For BI analysis, four independent Markov chain Monte Carlo Chains (MCMC) were run simultaneously for 10 ×10^6^ generations with sampling every 1000th generation) as implemented with MrBayes 3.1.2^38^. Two runs were conducted independently, and the first 25% of the tree discarded as burn-in. The ML was performed, under model GTR + G as evaluated in PartitionFinder^37^, with 100 random addition replicates and per partition branch lengths^39^ as implemented in RAXML v. 7.0.3^40^. The reliability of the ML tree was assessed by bootstrap analysis^41^ including 1000 replications. The resulting BI and ML trees were visualized using FigTree v1.4.2^42^. Bayesian Posterior Probabilities ≥ 0.95 for BI and bootstrap proportions ≥ 70% for ML were considered strongly supported. To visualize the relationships between haplotypes, a haplotype network was constructed using the median-joining algorithm^43^ implemented in Network 5.0.1.1 (www.fluxus-engineering.com).

To estimate the likely number of species units within *S. intermedius*, three different species delimitation approaches were employed:

In approach 1, species unit was assessed with TaxonDNA 1.8 with the ‘Cluster’ algorithm implemented in SpeciesIdentifier^44^. This method considers overlaps between the intra and interspecific variation, and the maximum pairwise distance within recognized putative species-level criterion should not exceed a given threshold. Species unit, herein termed clusters, are identified according to pairwise (uncorrected) distances for sequences within each cluster. We reduced the dataset to include the 31 unique haplotypes of *S. intermedius* previously identified. Incremental values ranged from 1.0% with an increase of 0.5% in each step to a maximum of 3.0%.

In approach 2, the automatic barcode gap discovery (ABGD)^45^ was performed on the online server (http://wwwabi.snv.jussieu.fr/public/abgd/) using all 220 sequences of *S. intermedius*. ABGD sorts the terminals into hypothetical species with calculated p-values based on the barcode gap. ABGD analyses used Kimura 2-parameter (K2P) and Jukes-Cantor (JC69) distances with setting parameters: Pmin = 0.001, Pmax = 0.2, relative gap width = 1.5 and Nb bins (for distance distribution) = 20, with the other parameters at default values.

In approach 3, PTP analyses were conducted on the bPTP web server (http://species.h-its.org/ptp/) using the RAxML tree of the unique haplotypes as input data (out-groups removed before analysis) with 100,000 MCMC generations, thinning set to 100, burn-in at 25% and performing a Bayesian search. The probability of each node to represent a species node was calculated using the maximum likelihood solution.

### Population genetic analyses and historical demography

Since genetic diversity is reflected by the measurement of nucleotide diversity (π) and haplotype diversity (h), we computed the number of haplotypes (H), haplotype diversity (h), nucleotide diversity (π) and mean number of pairwise differences (k) for each matriline using DnaSP. The historical demographics of the matrilines of *S. intermedius* were evaluated using arrays of statistics: First, mismatch distributions^46^ were calculated with Arlequin 3.5^47^ and used to examine signals of population expansion or stability over time. We assumed population stability would generate multimodal distribution, while expansion would imply unimodal pattern^48^. We compared observed distributions of nucleotide differences between pairs of haplotypes with those expected under spatial^49^ and demographic^47^ expansion models by using the generalized least square approach. In addition, we used the sum of squared deviations (SSD) as goodness-of-fit statistics for the observed and expected mismatch distributions, and the significance of fit for expansion model was tested, while the confidence intervals for the associated parameters estimates using 1000 bootstrap replicates were examined. Secondly, for the neutrality test, Tajima’s D^50^, Fu and Li’s D, and F^51^ tests were conducted for each matriline using Arlequin. Our assumption is that, if population sizes had been stable across time, Tajima’s D and Fu and Li’s D would be near zero. However, we assumed that significantly positive values would be expected in populations that experienced recent bottlenecks, and significantly negative values imply the recent population expansions^50,51^. We also computed Fs^52^ since causation is difficult to ascertain when Tajima’s D deviates significantly from zero. This statistic is particularly useful for detecting population expansions. We also assumed that a negative value of Fs implies recent population expansion or genetic hitchhiking, while a positive value results from a recent population bottleneck.

## Supplementary information

Figure S1.

Table S1.

Table S2.

Table S3.

Table S4.

## Data Availability

DNA sequences- GenBank Accession Nos MN509590 – MN509707; for each of the individual, details on locality information and GenBank Accession no. for its sequence data are shown in Table S1. All other data generated or analysed in this study are included in this published article (and its Supplementary Information files).
