# Supplementary material for: DNA Barcoding Silver Butter Catfish (Schilbe intermedius) Reveals Patterns of Mitochondrial Genetic Diversity Across African River Systems
Source: Sci Rep. 2020 Apr 27;10:7097. doi: 10.1038/s41598-020-63837-4 (PMC7184614; doi:10.1038/s41598-020-63837-4)
Supplement: Supplementary file 1 — Figure S1. [file 41598_2020_63837_MOESM1_ESM.pdf]

Title: DNA Barcoding Silver Butter Catfish (*Schilbe intermedius*) Reveals Patterns of Mitochondrial Genetic Diversity Across African River Systems.

Lotanna M. Nneji [1, 2, 17*], Adeniyi C. Adeola[1,2,17 *], Moshood K. Mustapha [3], Segun O. Oladipo[4], Chabi A. M. S. Djagoun[5], Ifeanyi C. Nneji[6], Babatunde E. Adedeji[7], Omotoso Olatunde[7], Adeola O. Ayoola[1], Agboola O. Okeyoyin[8], Odion O. Ikhimiukor[9], Galadima F. Useni[10], Oluyinka A. Iyiola[3], Emmanuel O. Faturoti[11], Moise M. Matouke[12], Wanze K. Ndifor[13], Yun-yu Wang[1], Jing Chen [14], Wen-Zhi Wang [1,14], Jolly B. Kachi[15], Obih A. Ugwumba[7], Adiaha A. A. Ugwumba[7], Christopher D. Nwani[16,*]

[1] State Key Laboratory of Genetic Resources and Evolution, Kunming Institute of Zoology, Chinese Academy of Sciences, Kunming 650223, China

[2] Sino-Africa Joint Research Centre, Chinese Academy of Sciences, Kunming, China

[3] Department of Zoology, Faculty of Life Sciences, University of Ilorin, Ilorin, Kwara State, Nigeria

[4] Department of Biosciences and Biotechnology, College of Pure and Applied Sciences, Kwara State University, Malete, Kwara State, Nigeria

[5] Laboratory of Applied Ecology, Faculty of Agronomic Sciences, University of Abomey-Calavi, Benin

[6] Department of Biological Science, Faculty of Sciences, University of Abuja, Abuja, Nigeria

[7] Department of Zoology, Faculty of Science, University of Ibadan, Ibadan, Oyo State, Nigeria

[8] National Park Service Headquarter, Federal Capital Territory, Abuja, Nigeria

[9] Department of Microbiology, Faculty of Science, University of Ibadan, Ibadan, Oyo State, Nigeria.

[10] Taraba State Polytechnic, Suntai, Taraba State, Nigeria

[11] Department of Aquaculture and Fisheries Management, Faculty of Agriculture, University of Ibadan, Ibadan, Oyo State, Nigeria.

[12] Department of Zoology, Faculty of Science, University of Douala, Douala, Cameroon.

[13] Department of Zoology, Faculty of Science, University of Dschang, Dschang, Cameroon.

[14] Wild Forensic Center, Kunming, China

[15] Department of Biological Sciences, Faculty of Sciences, Federal University Lokoja, Lokoja, Nigeria

[16] Department of Zoology and Environmental Biology, Faculty of Biological Sciences, University of Nigeria, Nsukka, Nigeria.

[17] These authors contributed equally to this work

*Correspondence: Lotanna Micah Nneji, lotannanneji@gmail.com; Adeniyi C. Adeola, chadeola@mail.kiz.ac.cn; Christopher D. Nwani, chris.nwani@unn.edu.ng

Figure S1: ABGD based partition of the complete data of *COI* sequences of *Schilbe intermedius* from African river systems based on (A) Kimura 2-parameter (K2P) and, (B) Jukes Cantor (JC69) distances. The initial partition is shown in yellow and recursive partition in red.

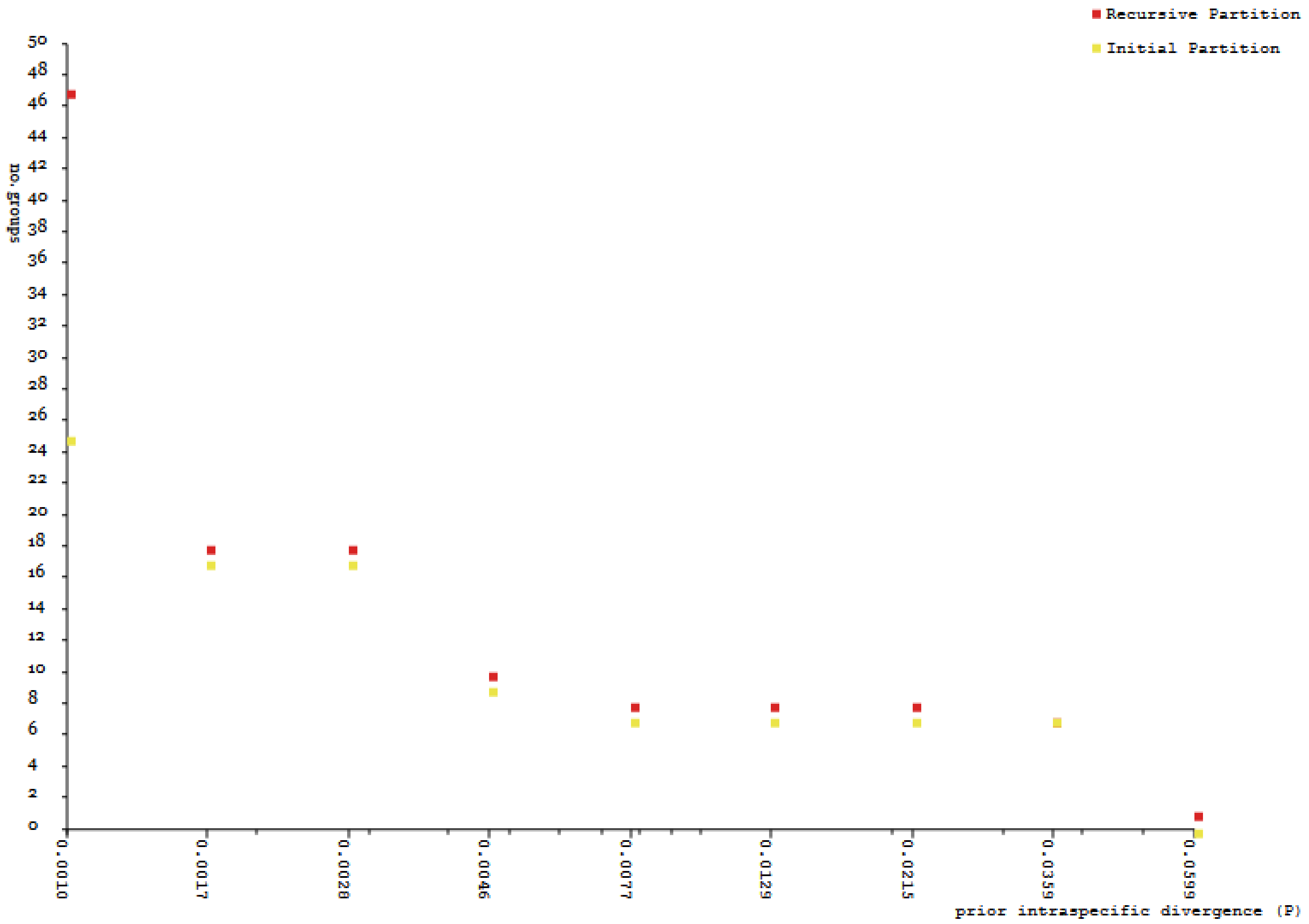

Recursive Partition
Initial Partition
no. groups
50
48
46
44
42
40
38
36
34
32
30
28
26
24
22
20
18
16
14
12
10
8
6
4
2
0
0.0010
0.0017
0.0028
0.0046
0.0077
0.0129
0.0215
0.0359
0.0599
prior intraspecific divergence (P)

(A)

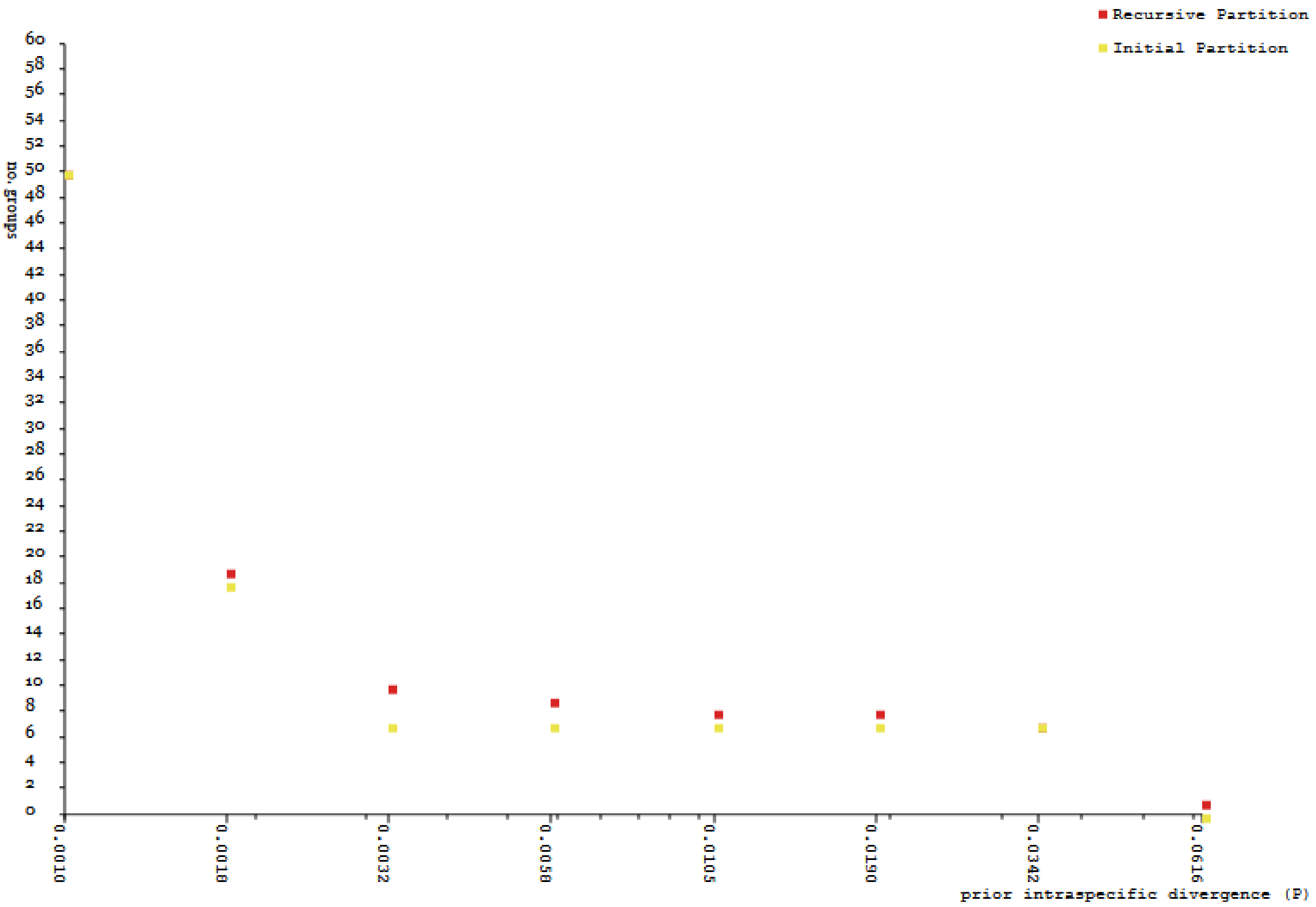
Recursive Partition
Initial Partition
no. groups
0
2
4
6
8
10
12
14
16
18
20
22
24
26
28
30
32
34
36
38
40
42
44
46
48
50
52
54
56
58
60
0.0010
0.0018
0.0032
0.0058
0.0105
0.0190
0.0342
0.0616
prior intraspecific divergence (P)

(B)
