# Supplementary material for: DNA Barcoding Silver Butter Catfish (Schilbe intermedius) Reveals Patterns of Mitochondrial Genetic Diversity Across African River Systems
Source: Sci Rep. 2020 Apr 27;10:7097. doi: 10.1038/s41598-020-63837-4 (PMC7184614; doi:10.1038/s41598-020-63837-4)
Supplement: Supplementary file 2 — Table S1. [file 41598_2020_63837_MOESM2_ESM.docx]

**Title**: DNA Barcoding Silver Butter Catfish (*Schilbe intermedius*) Reveals Patterns of Mitochondrial Genetic Diversity Across African River Systems.

Lotanna M. Nneji ^1, 2, 17*^, Adeniyi C. Adeola^1,2,17 *^, Moshood K. Mustapha ^3^, Segun O. Oladipo^4^, Chabi A. M. S. Djagoun^5^, Ifeanyi C. Nneji^6^, Babatunde E. Adedeji^7^, Omotoso Olatunde^7^, Adeola O. Ayoola^1^, Agboola O. Okeyoyin^8^, Odion O. Ikhimiukor^9^, Galadima F. Useni^10^, Oluyinka A. Iyiola^3^, Emmanuel O. Faturoti^11^, Moise M. Matouke^12^, Wanze K. Ndifor^13^, Yun-yu Wang^1^, Jing Chen ^14^, Wen-Zhi Wang ^1,14^, Jolly B. Kachi^15^, Obih A. Ugwumba^7^, Adiaha A. A. Ugwumba^7^, Christopher D. Nwani^16,*^

^1^ State Key Laboratory of Genetic Resources and Evolution, Kunming Institute of Zoology, Chinese Academy of Sciences, Kunming 650223, China

^2^ Sino-Africa Joint Research Centre, Chinese Academy of Sciences, Kunming, China

^3^ Department of Zoology, Faculty of Life Sciences, University of Ilorin, Ilorin, Kwara State, Nigeria

^4^ Department of Biosciences and Biotechnology, College of Pure and Applied Sciences, Kwara State University, Malete, Kwara State, Nigeria

^5^ Laboratory of Applied Ecology, Faculty of Agronomic Sciences, University of Abomey-Calavi, Benin

^6^ Department of Biological Science, Faculty of Sciences, University of Abuja, Abuja, Nigeria

^7^ Department of Zoology, Faculty of Science, University of Ibadan, Ibadan, Oyo State, Nigeria

^8^ National Park Service Headquarter, Federal Capital Territory, Abuja, Nigeria

^9^ Department of Microbiology, Faculty of Science, University of Ibadan, Ibadan, Oyo State, Nigeria.

^10^ Taraba State Polytechnic, Suntai, Taraba State, Nigeria

^11^ Department of Aquaculture and Fisheries Management, Faculty of Agriculture, University of Ibadan, Ibadan, Oyo State, Nigeria.

^12^ Department of Zoology, Faculty of Science, University of Douala, Douala, Cameroon.

^13^ Department of Zoology, Faculty of Science, University of Dschang, Dschang, Cameroon.

^14^ Wild Forensic Center, Kunming, China

^15^ Department of Biological Sciences, Faculty of Sciences, Federal University Lokoja, Lokoja, Nigeria

^16^ Department of Zoology and Environmental Biology, Faculty of Biological Sciences, University of Nigeria, Nsukka, Nigeria.

^17^ These authors contributed equally to this work

*Correspondence: Lotanna Micah Nneji, lotannanneji@gmail.com; Adeniyi C. Adeola, [chadeola@mail.kiz.ac.cn](mailto:chadeola@mail.kiz.ac.cn); Christopher D. Nwani, [chris.nwani@unn.edu.ng](mailto:chris.nwani@unn.edu.ng)

**Table S1: Sample Information of newly collected *Schilbe intermedius* from Nigeria and other sequences mined from BOLD and NCBI GenBank databases for the phylogenetic study**

| **Region** | **Ingroup** | **Species ID No/Voucher NO** | **Locality** | **BOLD ID** | **GenBank Accession Number** | **Reference** | **Haplogroup** | **Matrilineal Group** |
| --- | --- | --- | --- | --- | --- | --- | --- | --- |
| West Africa |  |  |  |  |  |  |  |  |
|  | *S. intermedius* 1 | ACA001 | Nigeria: Taraba, Donga Local Government Area |  | MN509590 | Current study | Hap_20 | E |
|  | *S. intermedius* 2 | ACA002 | Nigeria: Taraba, Donga Local Government Area |  | MN509591 | Current study | Hap_22 | E |
|  | *S. intermedius* 3 | ACA003 | Nigeria: Taraba, Donga Local Government Area |  | MN509592 | Current study | Hap_20 | E |
|  | *S. intermedius* 4 | ACA004 | Nigeria: Taraba, Donga Local Government Area |  | MN509593 | Current study | Hap_20 | E |
|  | *S. intermedius* 5 | ACA005 | Nigeria: Taraba, Donga Local Government Area |  | MN509594 | Current study | Hap_31 | E |
|  | *S. intermedius* 6 | ACA006 | Nigeria: Taraba, Donga Local Government Area |  | MN509595 | Current study | Hap_20 | E |
|  | *S. intermedius* 7 | ACA007 | Nigeria: Taraba, Donga Local Government Area |  | MN509596 | Current study | Hap_18 | E |
|  | *S. intermedius* 8 | ACA008 | Nigeria: Taraba, Donga Local Government Area |  | MN509597 | Current study | Hap_20 | E |
|  | *S. intermedius* 9 | ACA009 | Nigeria: Taraba, Donga Local Government Area |  | MN509598 | Current study | Hap_20 | E |
|  | *S. intermedius* 10 | ACA010 | Nigeria: Taraba, Donga Local Government Area |  | MN509599 | Current study | Hap_20 | E |
|  | *S. intermedius* 11 | ACA011 | Nigeria: Taraba, Takum Local Government Area |  | MN509600 | Current study | Hap_4 | A |
|  | *S. intermedius* 12 | ACA012 | Nigeria: Taraba, Takum Local Government Area |  | MN509601 | Current study | Hap_3 | A |
|  | *S. intermedius* 13 | ACA013 | Nigeria: Taraba, Takum Local Government Area |  | MN509602 | Current study | Hap_4 | A |
|  | *S. intermedius* 14 | ACA014 | Nigeria: Taraba, Takum Local Government Area |  | MN509603 | Current study | Hap_3 | A |
|  | *S. intermedius* 15 | ACA015 | Nigeria: Taraba, Takum Local Government Area |  | MN509604 | Current study | Hap_4 | A |
|  | *S. intermedius* 16 | ACA016 | Nigeria: Taraba, Takum Local Government Area |  | MN509605 | Current study | Hap_4 | A |
|  | *S. intermedius* 17 | ACA017 | Nigeria: Taraba, Takum Local Government Area |  | MN509606 | Current study | Hap_3 | A |
|  | *S. intermedius* 18 | ACA018 | Nigeria: Taraba, Takum Local Government Area |  | MN509607 | Current study | Hap_4 | A |
|  | *S. intermedius* 19 | ACA019 | Nigeria: Taraba, Takum Local Government Area |  | MN509608 | Current study | Hap_3 | A |
|  | *S. intermedius* 20 | ACA020 | Nigeria: Taraba, Takum Local Government Area |  | MN509609 | Current study | Hap_3 | A |
|  | *S. intermedius* 21 | ACA021 | Nigeria: Taraba, Takum Local Government Area |  | MN509610 | Current study | Hap_3 | A |
|  | *S. intermedius* 22 | ACA022 | Nigeria: Taraba, Takum Local Government Area |  | MN509611 | Current study | Hap_3 | A |
|  | *S. intermedius* 23 | ACA023 | Nigeria: Taraba, Takum Local Government Area |  | MN509612 | Current study | Hap_3 | A |
|  | *S. intermedius* 24 | ACA024 | Nigeria: Taraba, Takum Local Government Area |  | MN509613 | Current study | Hap_3 | A |
|  | *S. intermedius* 25 | ACA025 | Nigeria: Taraba, Takum Local Government Area |  | MN509614 | Current study | Hap_3 | A |
|  | *S. intermedius* 26 | SC001 | Nigeria: Kogi State, Lokoja |  | MN509615 | Current study | Hap_3 | A |
|  | *S. intermedius* 27 | SC002 | Nigeria: Kogi State, Lokoja |  | MN509616 | Current study | Hap_25 | E |
|  | *S. intermedius* 28 | SC003 | Nigeria: Kogi State, Lokoja |  | MN509617 | Current study | Hap_27 | A |
|  | *S. intermedius* 29 | SC004 | Nigeria: Kogi State, Lokoja |  | MN509618 | Current study | Hap_28 | A |
|  | *S. intermedius* 30 | SC006 | Nigeria: Kogi State, Lokoja |  | MN509619 | Current study | Hap_27 | A |
|  | *S. intermedius* 31 | SC007 | Nigeria: Kogi State, Lokoja |  | MN509620 | Current study | Hap_3 | A |
|  | *S. intermedius* 32 | SC007B | Nigeria: Taraba, Donga Local Government Area |  | MN509621 | Current study | Hap_31 | E |
|  | *S. intermedius* 33 | SC008 | Nigeria: Kogi State, Lokoja |  | MN509622 | Current study | Hap_4 | A |
|  | *S. intermedius* 34 | SC009 | Nigeria: Kogi State, Lokoja |  | MN509623 | Current study | Hap_27 | A |
|  | *S. intermedius* 35 | SC010 | Nigeria: Kogi State, Lokoja |  | MN509624 | Current study | Hap_23 | A |
|  | *S. intermedius* 36 | SC011 | Nigeria: Kogi State, Lokoja |  | MN509625 | Current study | Hap_3 | A |
|  | *S. intermedius* 37 | SC012 | Nigeria: Kogi State, Lokoja |  | MN509626 | Current study | Hap_3 | A |
|  | *S. intermedius* 38 | SC013 | Nigeria: Kogi State, Lokoja |  | MN509627 | Current study | Hap_24 | A |
|  | *S. intermedius* 39 | SC014 | Nigeria: Kogi State, Lokoja |  | MN509628 | Current study | Hap_3 | A |
|  | *S. intermedius* 40 | SC015 | Nigeria: Kogi State, Lokoja |  | MN509629 | Current study | Hap_25 | E |
|  | *S. intermedius* 41 | SC016 | Nigeria: Kogi State, Lokoja |  | MN509630 | Current study | Hap_3 | A |
|  | *S. intermedius* 42 | SC017 | Nigeria: Kogi State, Lokoja |  | MN509631 | Current study | Hap_26 | A |
|  | *S. intermedius* 43 | SC018 | Nigeria: Kogi State, Lokoja |  | MN509632 | Current study | Hap_3 | A |
|  | *S. intermedius* 44 | SC019 | Nigeria: Kogi State, Lokoja |  | MN509633 | Current study | Hap_3 | A |
|  | *S. intermedius* 45 | SC020 | Nigeria: Kogi State, Lokoja |  | MN509634 | Current study | Hap_3 | A |
|  | *S. intermedius* 46 | SC021 | Nigeria: Kogi State, Lokoja |  | MN509635 | Current study | Hap_25 | E |
|  | *S. intermedius* 47 | SC022 | Nigeria: Kogi State, Lokoja |  | MN509636 | Current study | Hap_25 | E |
|  | *S. intermedius* 48 | SC023 | Nigeria: Kogi State, Lokoja |  | MN509637 | Current study | Hap_3 | A |
|  | *S. intermedius* 49 | SC024 | Nigeria: Kogi State, Lokoja |  | MN509638 | Current study | Hap_25 | E |
|  | *S. intermedius* 50 | SC025 | Nigeria: Kogi State, Lokoja |  | MN509639 | Current study | Hap_25 | E |
|  | *S. intermedius* 51 | SC026 | Nigeria: Kogi State, Lokoja |  | MN509640 | Current study | Hap_3 | A |
|  | *S. intermedius* 52 | SC027 | Nigeria: Kogi State, Lokoja |  | MN509641 | Current study | Hap_3 | A |
|  | *S. intermedius* 53 | SC028 | Nigeria: Kogi State, Lokoja |  | MN509642 | Current study | Hap_3 | A |
|  | *S. intermedius* 54 | SC029 | Nigeria: Kogi State, Lokoja |  | MN509643 | Current study | Hap_25 | E |
|  | *S. intermedius* 55 | SC030 | Nigeria: Kogi State, Lokoja |  | MN509644 | Current study | Hap_26 | A |
|  | *S. intermedius* 56 | SC031 | Nigeria: Kogi State, Lokoja |  | MN509645 | Current study | Hap_25 | E |
|  | *S. intermedius* 57 | SC032 | Nigeria: Kogi State, Lokoja |  | MN509646 | Current study | Hap_27 | A |
|  | *S. intermedius* 58 | SC033 | Nigeria: Osun State, Ede |  | MN509647 | Current study | Hap_21 | A |
|  | *S. intermedius* 59 | SC034 | Nigeria: Osun State, Ede |  | MN509648 | Current study | Hap_21 | A |
|  | *S. intermedius* 60 | SC035 | Nigeria: Osun State, Ede |  | MN509649 | Current study | Hap_21 | A |
|  | *S. intermedius* 61 | SC036 | Nigeria: Osun State, Ede |  | MN509650 | Current study | Hap_21 | A |
|  | *S. intermedius* 62 | SC037 | Nigeria: Osun State, Ede |  | MN509651 | Current study | Hap_22 | E |
|  | *S. intermedius* 63 | SC038 | Nigeria: Osun State, Ede |  | MN509652 | Current study | Hap_3 | A |
|  | *S. intermedius* 064 | SC039 | Nigeria: Osun State, Ede |  | MN509653 | Current study | Hap_20 | E |
|  | *S. intermedius* 065 | SC040 | Nigeria: Oyo State, Ogbomosho |  | MN509654 | Current study | Hap_20 | E |
|  | *S. intermedius* 066 | SC041 | Nigeria: Oyo State, Ogbomosho |  | MN509655 | Current study | Hap_20 | E |
|  | *S. intermedius* 067 | SC042 | Nigeria: Oyo State, Ogbomosho |  | MN509656 | Current study | Hap_20 | E |
|  | *S. intermedius* 68 | SC043 | Nigeria: Oyo State, Ogbomosho |  | MN509657 | Current study | Hap_3 | A |
|  | *S. intermedius* 69 | SC044 | Nigeria: Oyo State, Ogbomosho |  | MN509658 | Current study | Hap_3 | A |
|  | *S. intermedius* 70 | SC045 | Nigeria: Oyo State, Ogbomosho |  | MN509659 | Current study | Hap_3 | A |
|  | *S. intermedius* 071 | SC047 | Nigeria: Oyo State, Ogbomosho |  | MN509660 | Current study | Hap_20 | E |
|  | *S. intermedius* 072 | SC048 | Nigeria: Oyo State, Ogbomosho |  | MN509661 | Current study | Hap_20 | E |
|  | *S. intermedius* 073 | SC050 | Nigeria: Oyo State, Ogbomosho |  | MN509662 | Current study | Hap_20 | E |
|  | *S. intermedius* 074 | SC051 | Nigeria: Oyo State, Ogbomosho |  | MN509663 | Current study | Hap_20 | E |
|  | *S. intermedius* 075 | SC052 | Nigeria: Oyo State, Ogbomosho |  | MN509664 | Current study | Hap_20 | E |
|  | *S. intermedius* 076 | SC053 | Nigeria: Oyo State, Ogbomosho |  | MN509665 | Current study | Hap_20 | E |
|  | *S. intermedius* 77 | SC054 | Nigeria: Oyo State, Ogbomosho |  | MN509666 | Current study | Hap_3 | A |
|  | *S. intermedius* 078 | SC055 | Nigeria: Kwara State, Moro |  | MN509667 | Current study | Hap_20 | E |
|  | *S. intermedius* 79 | SC056 | Nigeria: Kwara State, Moro |  | MN509668 | Current study | Hap_3 | A |
|  | *S. intermedius* 80 | SC057 | Nigeria: Kwara State, Moro |  | MN509669 | Current study | Hap_3 | A |
|  | *S. intermedius* 081 | SC058 | Nigeria: Kwara State, Moro |  | MN509670 | Current study | Hap_20 | E |
|  | *S. intermedius* 82 | SC059 | Nigeria: Kwara State, Moro |  | MN509671 | Current study | Hap_3 | A |
|  | *S. intermedius* 083 | SC060 | Nigeria: Kwara State, Moro |  | MN509672 | Current study | Hap_20 | E |
|  | *S. intermedius* 084 | SC061 | Nigeria: Kwara State, Moro |  | MN509673 | Current study | Hap_20 | E |
|  | *S. intermedius* 85 | SC062 | Nigeria: Kwara State, Moro |  | MN509674 | Current study | Hap_3 | A |
|  | *S. intermedius* 86 | SC063 | Nigeria: Kwara State, Moro |  | MN509675 | Current study | Hap_3 | A |
|  | *S. intermedius* 87 | SC064 | Nigeria: Kwara State, Moro |  | MN509676 | Current study | Hap_3 | A |
|  | *S. intermedius* 88 | SC065 | Nigeria: Kwara State, Afon |  | MN509677 | Current study | Hap_3 | A |
|  | *S. intermedius* 89 | SC066 | Nigeria: Kwara State, Afon |  | MN509678 | Current study | Hap_3 | A |
|  | *S. intermedius* 090 | SC067 | Nigeria: Kwara State, Afon |  | MN509679 | Current study | Hap_20 | E |
|  | *S. intermedius* 091 | SC068 | Nigeria: Kwara State, Afon |  | MN509680 | Current study | Hap_20 | E |
|  | *S. intermedius* 92 | SC069 | Nigeria: Kwara State, Afon |  | MN509681 | Current study | Hap_29 | A |
|  | *S. intermedius* 093 | SC070 | Nigeria: Kwara State, Afon |  | MN509682 | Current study | Hap_20 | E |
|  | *S. intermedius* 94 | SC071 | Nigeria: Kwara State, Afon |  | MN509683 | Current study | Hap_3 | A |
|  | *S. intermedius* 95 | SC072 | Nigeria: Kwara State, Afon |  | MN509684 | Current study | Hap_3 | A |
|  | *S. intermedius* 096 | SC073 | Nigeria: Kwara State, Afon |  | MN509685 | Current study | Hap_20 | E |
|  | *S. intermedius* 97 | SC074 | Nigeria: Kwara State, Afon |  | MN509686 | Current study | Hap_3 | A |
|  | *S. intermedius* 98 | SC075 | Nigeria: Kwara State, Afon |  | MN509687 | Current study | Hap_20 | E |
|  | *S. intermedius* 99 | SC076 | Nigeria: Kwara State, Afon |  | MN509688 | Current study | Hap_20 | E |
|  | *S. intermedius* 100 | SC077 | Nigeria: Kwara State, Afon |  | MN509689 | Current study | Hap_20 | E |
|  | *S. intermedius* 101 | SC078 | Nigeria: Kwara State, Afon |  | MN509690 | Current study | Hap_3 | A |
|  | *S. intermedius* 102 | SC079 | Nigeria: Kwara State, Afon |  | MN509691 | Current study | Hap_20 | E |
|  | *S. intermedius* 103 | SC080 | Nigeria: Kwara State, Afon |  | MN509692 | Current study | Hap_20 | E |
|  | *S. intermedius* 104 | SC081 | Nigeria: Kwara State, Afon |  | MN509693 | Current study | Hap_20 | E |
|  | *S. intermedius* 105 | SC082 | Nigeria: Kwara State, Afon |  | MN509694 | Current study | Hap_20 | E |
|  | *S. intermedius* 106 | SC083 | Nigeria: Kwara State, Afon |  | MN509695 | Current study | Hap_3 | A |
|  | *S. intermedius* 107 | SC084 | Nigeria: Kwara State, Afon |  | MN509696 | Current study | Hap_3 | A |
|  | *S. intermedius* 108 | SC085 | Nigeria: Kwara State, Afon |  | MN509697 | Current study | Hap_3 | A |
|  | *S. intermedius* 109 | SC086 | Nigeria: Kwara State, Afon |  | MN509698 | Current study | Hap_20 | E |
|  | *S. intermedius* 110 | SC087 | Nigeria: Kwara State, Afon |  | MN509699 | Current study | Hap_20 | E |
|  | *S. intermedius* 111 | SC088 | Nigeria: Kwara State, Afon |  | MN509700 | Current study | Hap_3 | A |
|  | *S. intermedius* 112 | SC089 | Nigeria: Kwara State, Afon |  | MN509701 | Current study | Hap_3 | A |
|  | *S. intermedius* 113 | SC090 | Nigeria: Kwara State, Afon |  | MN509702 | Current study | Hap_3 | A |
|  | *S. intermedius* 114 | SC091 | Nigeria: Kwara State, Afon |  | MN509703 | Current study | Hap_3 | A |
|  | *S. intermedius* 115 | SC092 | Nigeria: Kwara State, Afon |  | MN509704 | Current study | Hap_3 | A |
|  | *S. intermedius* 116 | SC093 | Nigeria: Kwara State, Afon |  | MN509705 | Current study | Hap_20 | E |
|  | *S. intermedius* 117 | SC094 | Nigeria: Kwara State, Afon |  | MN509706 | Current study | Hap_20 | E |
|  | *S. intermedius* 118 | SC095 | Nigeria: Kwara State, Afon |  | MN509707 | Current study | Hap_20 | E |
|  | *S. intermedius* 119 | BAFEN262-10 | Nigeria, Ebonyi, Afikpo | BOLD:AAL5704 | HM882934 | Nwani et al., 2011 | Hap_2 | A |
|  | *S. intermedius* 120 | BAFEN263-10 | Nigeria, Otuocha | BOLD:AAL5704 | HM882935 | Nwani et al., 2011 | Hap_3 | A |
|  | *S. intermedius* 121 | BAFEN264-10 | Nigeria, Anambra | BOLD:AAL5704 | HM882936 | Nwani et al., 2011 | Hap_3 | A |
|  | *S. intermedius* 122 | BAFEN268-10 | Nigeria, Anambra | BOLD:AAL5704 | HM882940 | Nwani et al., 2011 | Hap_3 | A |
|  | *S. intermedius* 123 | BAFEN269-10 | Nigeria, Anambra | BOLD:AAL5704 | HM882941 | Nwani et al., 2011 | Hap_3 | A |
|  | *S. intermedius* 124 | BAFEN272-10 | Nigeria, Ebonyi, Abakiliki | BOLD:AAL5704 | HM882944 | Nwani et al., 2011 | Hap_2 | A |
|  | *S. intermedius* 125 | BAFEN273-10 | Nigeria, Enugu, Abakaliki | BOLD:AAL5704 | HM882945 | Nwani et al., 2011 | Hap_2 | A |
|  | *S. intermedius* 126 | BAFEN274-10 | Nigeria, Enugu, Abakaliki | BOLD:AAL5704 | HM882946 | Nwani et al., 2011 | Hap_2 | A |
|  | *S. intermedius* 127 | BAFEN275-10 | Nigeria, Ebonyi, Abakiliki | BOLD:AAL5704 | HM882947 | Nwani et al., 2011 | Hap_2 | A |
|  | *S. intermedius* 128 | BAFEN276-10 | Nigeria, Ebonyi, Abakiliki | BOLD:AAL5704 | HM882948 | Nwani et al., 2011 | Hap_2 | A |
|  | *S. intermedius* 129 | BAFEN296-10 | Nigeria, Otuocha | BOLD:AAL5704 | HM882962 | Nwani et al., 2011 | Hap_4 | A |
|  | *S. intermedius* 130 | YLMC007 | Nigeria: Kwara State, Oyun |  | MG824640 | Iyiola et al., 2018 | Hap_20 | E |
|  | *S. intermedius* 131 | YLMC009 | Nigeria: Kwara State, Asa reservoir |  | MG824641 | Iyiola et al., 2018 | Hap_3 | A |
|  | *S. intermedius* 132 | YLMC016 | Nigeria: Kwara State, Asa reservoir |  | MG824642 | Iyiola et al., 2018 | Hap_20 | E |
|  | *S. intermedius* 133 | YLMC017 | Nigeria: Kwara State, Asa reservoir |  | MG824643 | Iyiola et al., 2018 | Hap_30 | E |
|  | *S. intermedius* 134 | YLMC034 | Nigeria: Kwara State, Kwara State, Awon River |  | MG824646 | Iyiola et al., 2018 | Hap_20 | E |
|  | *S. intermedius* 135 | YLMC049 | Nigeria |  | MG824649 | Iyiola et al., 2018 | Hap_3 | A |
|  | *S. intermedius* 136 | YLMC271 | Nigeria: Kwara State, Jebba HEP Downstream |  | MG824644 | Iyiola et al., 2018 | Hap_20 | E |
|  | *S. intermedius* 137 | YLMC285 | Nigeria: Niger State, Jebba HEP Upstream |  | MG824645 | Iyiola et al., 2018 | Hap_3 | A |
|  | *S. intermedius* 138 | YLMC450 | Nigeria: Kwara State, Kwara State, Asa-Laduba |  | MG824647 | Iyiola et al., 2018 | Hap_20 | E |
|  | *S. intermedius* 139 | YLMC451 | Nigeria: Kwara State, Kwara State, Asa-Laduba |  | MG824648 | Iyiola et al., 2018 | Hap_20 | E |
| Central Africa |  |  |  |  |  |  |  |  |
|  | *S. intermedius* 140 | AMNHI489-12 | DRC, Kinshasa, Nsele River | BOLD:ABW1933 |  | BOLD Database | Hap_1 | G |
|  | *S. intermedius* 141 | AMNHI711-12 | DRC, Kinshasa, Nsele River | BOLD:ABW1933 |  | BOLD Database | Hap_5 | G |
|  | *S. intermedius* 142 | DCF653-15 | DRC, Middle Congo River | BOLD:ABW1933 | KT193393 | Decru et al., 2016 | Hap_19 | G |
|  | *S. intermedius* 143 | DCF701-15 | DRC, Upper Congo River | BOLD:ACV4265 | KT193441 | Decru et al., 2016 | Hap_7 | F |
|  | *S. intermedius* 144 | GBMIN120836-17 | DRC, Congo River | BOLD:ABW1933 | KX186316 | BOLD Database | Hap_5 | G |
|  | *S. intermedius* 145 | GBMIN120884-17 | DRC, Congo River | BOLD:ABW1933 | KX186544 | BOLD Database | Hap_5 | G |
|  | *S. intermedius* 146 | GBMIN131023-17 | DRC, Congo River | BOLD:ABW1933 | KX186086 | BOLD Database | Hap_1 | G |
|  | *S. intermedius* 147 | GBMIN131026-17 | DRC, Congo River | BOLD:ABW1933 | KX186104 | BOLD Database | Hap_9 | G |
|  | *S. intermedius* 148 | GBMIN131083-17 | DRC, Congo River | BOLD:ABW1933 | KX186317 | BOLD Database | Hap_5 | G |
|  | *S. intermedius* 149 | GBMIN131084-17 | DRC, Congo River | BOLD:ABW1933 | KX186318 | BOLD Database | Hap_1 | G |
|  | *S. intermedius* 150 | GBMIN131085-17 | DRC, Congo River | BOLD:ABW1933 | KX186323 | BOLD Database | Hap_5 | G |
|  | *S. intermedius* 151 | AMNHI490-12 | DRC, Nsele River | BOLD:ABW1933 |  | BOLD Database | Hap_1 | G |
|  | *S. intermedius* 152 | DCF373-15 | DRC, Itimbiri River | BOLD:ABW1933 | KT193113 | Decru et al., 2016 | Hap_5 | G |
|  | *S. intermedius* 153 | DCF374-15 | DRC, Itimbiri River | BOLD:ABW1933 | KT193114 | Decru et al., 2016 | Hap_5 | G |
|  | *S. intermedius* 154 | DCF654-15 | DRC, Middle Congo River | BOLD:ABW1933 | KT193394 | Decru et al., 2016 | Hap_6 | G |
|  | *S. intermedius* 155 | GBMIN120823-17 | DRC, Congo River | BOLD:ABW1933 | KX186272 | BOLD Database | Hap_1 | G |
|  | *S. intermedius* 156 | GBMIN131122-17 | DRC, Congo River | BOLD:ABW1933 | KX186543 | BOLD Database | Hap_10 | G |
|  | *S. intermedius* 157 | GBMIN131123-17 | DRC, Congo River | BOLD:ABW1933 | KX186547 | BOLD Database | Hap_1 | G |
| East Africa |  |  |  |  |  |  |  |  |
|  | *S. intermedius* 158 | DOFKA340-08 | Kenya, Nyanza, Lake Victoria Basin | BOLD:AAD0084 |  | BOLD Database | Hap_8 | D |
|  | *S. intermedius* 159 | DOFKA360-08 | Kenya, Nyanza, Lake Victoria Basin | BOLD:AAD0084 |  | BOLD Database | Hap_8 | D |
|  | *S. intermedius* 160 | DOFKA367-08 | Kenya, Nyanza, Lake Victoria Basin | BOLD:AAD0084 |  | BOLD Database | Hap_8 | D |
|  | *S. intermedius* 161 | DOFKA372-08 | Kenya, Nyanza, Lake Victoria Basin | BOLD:AAD0084 |  | BOLD Database | Hap_8 | D |
|  | *S. intermedius* 162 | HVDBF086-10 | Kenya, Kipende | BOLD:AAO5173 |  | BOLD Database | Hap_15 | C |
|  | *S. intermedius* 163 | HVDBF087-10 | Kenya, Kipende | BOLD:AAO5173 |  | BOLD Database | Hap_14 | C |
|  | *S. intermedius* 164 | HVDBF089-10 | Kenya, Wenje | BOLD:AAO5173 |  | BOLD Database | Hap_15 | C |
|  | *S. intermedius* 165 | HVDBF567-12 | Kenya, Coast, Wenje, Tana River | BOLD:AAO5173 |  | BOLD Database | Hap_15 | C |
|  | *S. intermedius* 166 | HVDBF568-12 | Kenya, Coast, Wenje, Tana River | BOLD:AAO5173 |  | BOLD Database | Hap_15 | C |
|  | *S. intermedius* 167 | HVDBF570-12 | Kenya, Coast, Wenje, Tana River | BOLD:AAO5173 |  | BOLD Database | Hap_15 | C |
|  | *S. intermedius* 168 | DOFKA182-08 | Kenya, Nyanza, Lake Victoria Basin | BOLD:AAD0084 |  | BOLD Database | Hap_8 | D |
|  | *S. intermedius* 169 | HVDBF088-10 | Kenya, Wenje | BOLD:AAO5173 |  | BOLD Database | Hap_15 | C |
|  | *S. intermedius* 170 | HVDBF566-12 | Kenya, Coast, Wenje, Tana River | BOLD:AAO5173 |  | BOLD Database | Hap_15 | C |
|  | *S. intermedius* 171 | HVDBF569-12 | Kenya, Coast, Wenje, Tana River | BOLD:AAO5173 |  | BOLD Database | Hap_17 | C |
| Botswana |  |  |  |  |  |  |  |  |
|  | *S. intermedius* 172 | HVDB038-09 | Botswana, North West | BOLD:ACA7322 |  | BOLD Database | Hap_11 | B |
|  | *S. intermedius* 173 | HVDBF032-10 | Botswana, Lesedeng | BOLD:ACA7322 |  | BOLD Database | Hap_13 | B |
|  | *S. intermedius* 174 | HVDBF033-10 | Botswana, Lesedeng | BOLD:ACA7322 |  | BOLD Database | Hap_11 | B |
|  | *S. intermedius* 175 | HVDBF034-10 | Botswana, Lesedeng | BOLD:ACA7322 |  | BOLD Database | Hap_13 | B |
|  | *S. intermedius* 176 | HVDB039-09 | Botswana, North West | BOLD:ACA7322 |  | BOLD Database | Hap_11 | B |
|  | *S. intermedius* 177 | HVDBF048-10 | Botswana | BOLD:ACA7322 |  | BOLD Database | Hap_11 | B |
| Namibia |  |  |  |  |  |  |  |  |
|  | *S. intermedius* 178 | HVDBF242-11 | Namibia, Cunene Mouth | BOLD:ACA7322 |  | BOLD Database | Hap_16 | B |
|  | *S. intermedius* 179 | HVDBF243-11 | Namibia, Cunene Mouth | BOLD:ACA7322 |  | BOLD Database | Hap_16 | B |
|  | *S. intermedius* 180 | HVDBF251-11 | Namibia, Cunene Mouth | BOLD:ACA7322 |  | BOLD Database | Hap_16 | B |
|  | *S. intermedius* 181 | HVDBF252-11 | Namibia, Cunene Mouth | BOLD:ACA7322 |  | BOLD Database | Hap_16 | B |
|  | *S. intermedius* 182 | HVDBF744-12 | Namibia, Kunene, Kaokoland, Cunene River | BOLD:ACA7322 |  | BOLD Database | Hap_16 | B |
|  | *S. intermedius* 183 | HVDBF747-12 | Namibia, Kunene, Kaokoland, Cunene River | BOLD:ACA7322 |  | BOLD Database | Hap_16 | B |
|  | *S. intermedius* 184 | HVDBF250-11 | Namibia, Cunene Mouth | BOLD:ACA7322 |  | BOLD Database | Hap_16 | B |
| Swaziland |  |  |  |  |  |  |  |  |
|  | *S. intermedius* 185 | HVDBM946-12 | Swaziland, Sandriver Dam | BOLD:ACA7295 |  | BOLD Database | Hap_12 | B |
|  | *S. intermedius* 186 | HVDBF666-12 | Swaziland, Sandriver Dam | BOLD:ACA7295 |  | BOLD Database | Hap_12 | B |
|  | *S. intermedius* 187 | HVDBF667-12 | Swaziland, Sandriver Dam | BOLD:ACA7295 |  | BOLD Database | Hap_12 | B |
|  | *S. intermedius* 188 | HVDBF668-12 | Swaziland, Sandriver Dam | BOLD:ACA7295 |  | BOLD Database | Hap_12 | B |
|  | *S. intermedius* 189 | HVDBF671-12 | Swaziland, Sandriver Dam | BOLD:ACA7295 |  | BOLD Database | Hap_12 | B |
|  | *S. intermedius* 190 | HVDBF673-12 | Swaziland, Sandriver Dam | BOLD:ACA7295 |  | BOLD Database | Hap_12 | B |
|  | *S. intermedius* 191 | HVDBF674-12 | Swaziland, Sandriver Dam | BOLD:ACA7295 |  | BOLD Database | Hap_12 | B |
|  | *S. intermedius* 192 | HVDBF676-12 | Swaziland, Sandriver Dam | BOLD:ACA7295 |  | BOLD Database | Hap_12 | B |
|  | *S. intermedius* 193 | HVDBF677-12 | Swaziland, Sandriver Dam | BOLD:ACA7295 |  | BOLD Database | Hap_12 | B |
|  | *S. intermedius* 194 | HVDBF678-12 | Swaziland, Sandriver Dam | BOLD:ACA7295 |  | BOLD Database | Hap_12 | B |
|  | *S. intermedius* 195 | HVDBF679-12 | Swaziland, Sandriver Dam | BOLD:ACA7295 |  | BOLD Database | Hap_12 | B |
|  | *S. intermedius* 196 | HVDBF681-12 | Swaziland, Sandriver Dam | BOLD:ACA7295 |  | BOLD Database | Hap_12 | B |
|  | *S. intermedius* 197 | HVDBF669-12 | Swaziland, Sandriver Dam | BOLD:ACA7295 |  | BOLD Database | Hap_12 | B |
|  | *S. intermedius* 198 | HVDBF670-12 | Swaziland, Sandriver Dam | BOLD:ACA7295 |  | BOLD Database | Hap_12 | B |
|  | *S. intermedius* 199 | HVDBF672-12 | Swaziland, Sandriver Dam | BOLD:ACA7295 |  | BOLD Database | Hap_12 | B |
|  | *S. intermedius* 200 | HVDBF675-12 | Swaziland, Sandriver Dam | BOLD:ACA7295 |  | BOLD Database | Hap_12 | B |
|  | *S. intermedius* 201 | HVDBF680-12 | Swaziland, Sandriver Dam | BOLD:ACA7295 |  | BOLD Database | Hap_12 | B |
|  | *S. intermedius* 202 | HVDBM947-12 | Swaziland, Sandriver Dam | BOLD:ACA7295 |  | BOLD Database | Hap_12 | B |
|  | *S. intermedius* 203 | HVDBM948-12 | Swaziland, Sandriver Dam | BOLD:ACA7295 |  | BOLD Database | Hap_12 | B |
|  | *S. intermedius* 204 | HVDBM949-12 | Swaziland, Sandriver Dam | BOLD:ACA7295 |  | BOLD Database | Hap_12 | B |
| South Africa |  |  |  |  |  |  |  |  |
|  | *S. intermedius* 205 | HVDB267-10 | South Africa, Mpumalanga | BOLD:ACA7295 |  | BOLD Database | Hap_12 | B |
|  | *S. intermedius* 206 | HVDB268-10 | South Africa, Mpumalanga | BOLD:ACA7295 |  | BOLD Database | Hap_12 | B |
|  | *S. intermedius* 207 | HVDB307-10 | South Africa, Mpumalanga | BOLD:ACA7295 |  | BOLD Database | Hap_12 | B |
|  | *S. intermedius* 208 | HVDBF067-10 | South Africa, KwaZulu-Natal | BOLD:ACA7295 |  | BOLD Database | Hap_12 | B |
|  | *S. intermedius* 209 | HVDBF069-10 | South Africa, KwaZulu-Natal | BOLD:ACA7295 |  | BOLD Database | Hap_12 | B |
|  | *S. intermedius* 210 | HVDBF090-10 | South Africa, KwaZulu-Natal | BOLD:ACA7295 |  | BOLD Database | Hap_12 | B |
|  | *S. intermedius* 211 | HVDBF091-10 | South Africa, KwaZulu-Natal | BOLD:ACA7295 |  | BOLD Database | Hap_12 | B |
|  | *S. intermedius* 212 | HVDBF092-10 | South Africa, KwaZulu-Natal | BOLD:ACA7295 |  | BOLD Database | Hap_12 | B |
|  | *S. intermedius* 213 | HVDBF094-10 | South Africa, KwaZulu-Natal | BOLD:ACA7295 |  | BOLD Database | Hap_12 | B |
|  | *S. intermedius* 214 | HVDB208-10 | South Africa, Mpumalanga | BOLD:ACA7295 |  | BOLD Database | Hap_12 | B |
|  | *S. intermedius* 215 | HVDB209-10 | South Africa, Mpumalanga | BOLD:ACA7295 |  | BOLD Database | Hap_12 | B |
|  | *S. intermedius* 216 | HVDBF066-10 | South Africa, KwaZulu-Natal, Ndumo Causeway | BOLD:ACA7295 |  | BOLD Database | Hap_12 | B |
|  | *S. intermedius* 217 | HVDBF068-10 | South Africa, KwaZulu-Natal, Ndumo Causeway | BOLD:ACA7295 |  | BOLD Database | Hap_12 | B |
|  | *S. intermedius* 218 | HVDBF093-10 | South Africa, KwaZulu-Natal, Ndumo Causeway | BOLD:ACA7295 |  | BOLD Database | Hap_12 | B |
|  | *S. intermedius* 219 | HVDB266-10 | South Africa, Mpumalanga, Arabie Dam | BOLD:ACA7295 |  | BOLD Database | Hap_13 | B |
|  | **Outgroup** |  |  |  |  |  |  |  |
|  | *Schilbe mystus* |  |  |  | HM882942 | Nwani et al., 2011 |  |  |
|  | *Schilbe multitaeniatus* | |  |  | MK074641 | Sonet et al., 2018 |  |  |
|  | *Schilbe grenfelli* |  |  |  | MK074630 | Sonet et al., 2018 |  |  |
|  | *Schilbe marmoratus* | |  |  | MK074638 | Sonet et al., 2018 |  |  |
|  | *Schilbe zairensis* |  |  |  | MK074643 | Sonet et al., 2018 |  |  |

**References**

1. BOLD Database: http://www.boldsystems.org/
2. Decru, E. *et al.* Taxonomic challenges in freshwater fishes: a mismatch between morphology and DNA barcoding in fish of the north-eastern part of the Congo basin. *Mol Ecol Resour* **16**, 342–352, <https://doi.org/10.1111/1755-0998.12445> (2016).
3. Iyiola, O. A. *et al*. DNA barcoding of Economically Important Freshwater Fish Species from North – Central Nigeria Uncovers Cryptic Diversity. *Ecol Evol* **8** (14), 6932–6951, https://doi.org/10.1002/ece3.4210 (2018).
4. Nwani, C. D. *et al.* DNA barcoding discriminates freshwater fishes from southeastern Nigeria and provides river system-level phylogeographic resolution within some species. *Mitochondrial DNA* **22** (suppl 1), 43–51, https://doi.org/10.3109/19401736.2010.536537 (2011).
5. Sonet, G. *et al.* DNA barcoding fishes from the Congo and the Lower Guinean provinces: Assembling a reference library for poorly inventoried fauna.  *Mol Ecol Res* **19** (3), 728 – 743, <https://doi.org/10.1111/1755-0998.12983> (2018).
