# Supplementary material for: DNA Barcoding Silver Butter Catfish (Schilbe intermedius) Reveals Patterns of Mitochondrial Genetic Diversity Across African River Systems
Source: Sci Rep. 2020 Apr 27;10:7097. doi: 10.1038/s41598-020-63837-4 (PMC7184614; doi:10.1038/s41598-020-63837-4)
Supplement: Supplementary file 3 — Table S2. [file 41598_2020_63837_MOESM3_ESM.docx]

**Title**: DNA Barcoding Silver Butter Catfish (*Schilbe intermedius*) Reveals Patterns of Mitochondrial Genetic Diversity Across African River Systems.

Lotanna M. Nneji ^1, 2, 17*^, Adeniyi C. Adeola^1,2,17 *^, Moshood K. Mustapha ^3^, Segun O. Oladipo^4^, Chabi A. M. S. Djagoun^5^, Ifeanyi C. Nneji^6^, Babatunde E. Adedeji^7^, Omotoso Olatunde^7^, Adeola O. Ayoola^1^, Agboola O. Okeyoyin^8^, Odion O. Ikhimiukor^9^, Galadima F. Useni^10^, Oluyinka A. Iyiola^3^, Emmanuel O. Faturoti^11^, Moise M. Matouke^12^, Wanze K. Ndifor^13^, Yun-yu Wang^1^, Jing Chen ^14^, Wen-Zhi Wang ^1,14^, Jolly B. Kachi^15^, Obih A. Ugwumba^7^, Adiaha A. A. Ugwumba^7^, Christopher D. Nwani^16,*^

^1^ State Key Laboratory of Genetic Resources and Evolution, Kunming Institute of Zoology, Chinese Academy of Sciences, Kunming 650223, China

^2^ Sino-Africa Joint Research Centre, Chinese Academy of Sciences, Kunming, China

^3^ Department of Zoology, Faculty of Life Sciences, University of Ilorin, Ilorin, Kwara State, Nigeria

^4^ Department of Biosciences and Biotechnology, College of Pure and Applied Sciences, Kwara State University, Malete, Kwara State, Nigeria

^5^ Laboratory of Applied Ecology, Faculty of Agronomic Sciences, University of Abomey-Calavi, Benin

^6^ Department of Biological Science, Faculty of Sciences, University of Abuja, Abuja, Nigeria

^7^ Department of Zoology, Faculty of Science, University of Ibadan, Ibadan, Oyo State, Nigeria

^8^ National Park Service Headquarter, Federal Capital Territory, Abuja, Nigeria

^9^ Department of Microbiology, Faculty of Science, University of Ibadan, Ibadan, Oyo State, Nigeria.

^10^ Taraba State Polytechnic, Suntai, Taraba State, Nigeria

^11^ Department of Aquaculture and Fisheries Management, Faculty of Agriculture, University of Ibadan, Ibadan, Oyo State, Nigeria.

^12^ Department of Zoology, Faculty of Science, University of Douala, Douala, Cameroon.

^13^ Department of Zoology, Faculty of Science, University of Dschang, Dschang, Cameroon.

^14^ Wild Forensic Center, Kunming, China

^15^ Department of Biological Sciences, Faculty of Sciences, Federal University Lokoja, Lokoja, Nigeria

^16^ Department of Zoology and Environmental Biology, Faculty of Biological Sciences, University of Nigeria, Nsukka, Nigeria.

^17^ These authors contributed equally to this work

*Correspondence: Lotanna Micah Nneji, lotannanneji@gmail.com; Adeniyi C. Adeola, [chadeola@mail.kiz.ac.cn](mailto:chadeola@mail.kiz.ac.cn); Christopher D. Nwani, [chris.nwani@unn.edu.ng](mailto:chris.nwani@unn.edu.ng)

**Table S2:** Clustering Analyses of 31 Unique Haplotypes of *Schilbe intermedius* performed in SpeciesIdentifier

Threshold: 1% – 3.0%

| Cluster | Number of Sequences | Matrilineal Group | Region |
| --- | --- | --- | --- |
| Cluster 1 | 6 | Matriline G | Central Africa-2 |
| Cluster 2 | 10 | Matriline A | West Africa_1 |
| Cluster 3 | 1 | Matriline D | East Africa-2 |
| Cluster 4 | 1 | Matriline F | Central Africa-1 |
| Cluster 5 | 4 | Matriline B | South Africa |
| Cluster 6 | 3 | Matriline C | East Africa-1 |
| Cluster 7 | 6 | Matriline E | West Africa_2 |

Matrilineal group represents matrilines identified by the phylogenetic analyses
