# Supplementary material for: DNA Barcoding Silver Butter Catfish (Schilbe intermedius) Reveals Patterns of Mitochondrial Genetic Diversity Across African River Systems
Source: Sci Rep. 2020 Apr 27;10:7097. doi: 10.1038/s41598-020-63837-4 (PMC7184614; doi:10.1038/s41598-020-63837-4)
Supplement: Supplementary file 4 — Table S3. [file 41598_2020_63837_MOESM4_ESM.docx]

**Title**: DNA Barcoding Silver Butter Catfish (*Schilbe intermedius*) Reveals Patterns of Mitochondrial Genetic Diversity Across African River Systems.

Lotanna M. Nneji ^1, 2, 17*^, Adeniyi C. Adeola^1,2,17 *^, Moshood K. Mustapha ^3^, Segun O. Oladipo^4^, Chabi A. M. S. Djagoun^5^, Ifeanyi C. Nneji^6^, Babatunde E. Adedeji^7^, Omotoso Olatunde^7^, Adeola O. Ayoola^1^, Agboola O. Okeyoyin^8^, Odion O. Ikhimiukor^9^, Galadima F. Useni^10^, Oluyinka A. Iyiola^3^, Emmanuel O. Faturoti^11^, Moise M. Matouke^12^, Wanze K. Ndifor^13^, Yun-yu Wang^1^, Jing Chen ^14^, Wen-Zhi Wang ^1,14^, Jolly B. Kachi^15^, Obih A. Ugwumba^7^, Adiaha A. A. Ugwumba^7^, Christopher D. Nwani^16,*^

^1^ State Key Laboratory of Genetic Resources and Evolution, Kunming Institute of Zoology, Chinese Academy of Sciences, Kunming 650223, China

^2^ Sino-Africa Joint Research Centre, Chinese Academy of Sciences, Kunming, China

^3^ Department of Zoology, Faculty of Life Sciences, University of Ilorin, Ilorin, Kwara State, Nigeria

^4^ Department of Biosciences and Biotechnology, College of Pure and Applied Sciences, Kwara State University, Malete, Kwara State, Nigeria

^5^ Laboratory of Applied Ecology, Faculty of Agronomic Sciences, University of Abomey-Calavi, Benin

^6^ Department of Biological Science, Faculty of Sciences, University of Abuja, Abuja, Nigeria

^7^ Department of Zoology, Faculty of Science, University of Ibadan, Ibadan, Oyo State, Nigeria

^8^ National Park Service Headquarter, Federal Capital Territory, Abuja, Nigeria

^9^ Department of Microbiology, Faculty of Science, University of Ibadan, Ibadan, Oyo State, Nigeria.

^10^ Taraba State Polytechnic, Suntai, Taraba State, Nigeria

^11^ Department of Aquaculture and Fisheries Management, Faculty of Agriculture, University of Ibadan, Ibadan, Oyo State, Nigeria.

^12^ Department of Zoology, Faculty of Science, University of Douala, Douala, Cameroon.

^13^ Department of Zoology, Faculty of Science, University of Dschang, Dschang, Cameroon.

^14^ Wild Forensic Center, Kunming, China

^15^ Department of Biological Sciences, Faculty of Sciences, Federal University Lokoja, Lokoja, Nigeria

^16^ Department of Zoology and Environmental Biology, Faculty of Biological Sciences, University of Nigeria, Nsukka, Nigeria.

^17^ These authors contributed equally to this work

*Correspondence: Lotanna Micah Nneji, lotannanneji@gmail.com; Adeniyi C. Adeola, [chadeola@mail.kiz.ac.cn](mailto:chadeola@mail.kiz.ac.cn); Christopher D. Nwani, [chris.nwani@unn.edu.ng](mailto:chris.nwani@unn.edu.ng)

**Table S3:** Results of the Automatic Barcode Gap Discovery (ABGD) analyses using Kimura 2-parameter (K2P) and Jukes Cantor (JC69) distances

*Parameters*

Pmin = 0.001

Pmax = 0.2

Relative gap width = 1.5

Nb bins (for distance distribution) = 20

*Kimura 80 distance*
Partition 1 : found 47 groups (prior maximal distance P= 0.001000)
Partition 2 : found 18 groups (prior maximal distance P= 0.001802)
Partition 3 : found 12 groups (prior maximal distance P= 0.003246)
Partition 4 : found 10 groups (prior maximal distance P= 0.005848)
Partition 5 : found 8 groups (prior maximal distance P= 0.010536)
Partition 6 : found 8 groups (prior maximal distance P= 0.018982)
Partition 7 : found 6 groups (prior maximal distance P= 0.034200)

*Jukes Cantor 69 distance*

Partition 1 : found 50 groups (prior maximal distance P= 0.001000)
Partition 2 : found 19 groups (prior maximal distance P= 0.001802)
Partition 3 : found 10 groups (prior maximal distance P= 0.003246)
Partition 4 : found 9 groups (prior maximal distance P= 0.005848)
Partition 5 : found 8 groups (prior maximal distance P= 0.010536)
Partition 6 : found 8 groups (prior maximal distance P= 0.018982)
Partition 7 : found 6 groups (prior maximal distance P= 0.034200)
