# Supplementary material for: DNA Barcoding Silver Butter Catfish (Schilbe intermedius) Reveals Patterns of Mitochondrial Genetic Diversity Across African River Systems
Source: Sci Rep. 2020 Apr 27;10:7097. doi: 10.1038/s41598-020-63837-4 (PMC7184614; doi:10.1038/s41598-020-63837-4)
Supplement: Supplementary file 5 — Table S4. [file 41598_2020_63837_MOESM5_ESM.docx]

**Title**: DNA Barcoding Silver Butter Catfish (*Schilbe intermedius*) Reveals Patterns of Mitochondrial Genetic Diversity Across African River Systems.

Lotanna M. Nneji ^1, 2, 17*^, Adeniyi C. Adeola^1,2,17 *^, Moshood K. Mustapha ^3^, Segun O. Oladipo^4^, Chabi A. M. S. Djagoun^5^, Ifeanyi C. Nneji^6^, Babatunde E. Adedeji^7^, Omotoso Olatunde^7^, Adeola O. Ayoola^1^, Agboola O. Okeyoyin^8^, Odion O. Ikhimiukor^9^, Galadima F. Useni^10^, Oluyinka A. Iyiola^3^, Emmanuel O. Faturoti^11^, Moise M. Matouke^12^, Wanze K. Ndifor^13^, Yun-yu Wang^1^, Jing Chen ^14^, Wen-Zhi Wang ^1,14^, Jolly B. Kachi^15^, Obih A. Ugwumba^7^, Adiaha A. A. Ugwumba^7^, Christopher D. Nwani^16,*^

^1^ State Key Laboratory of Genetic Resources and Evolution, Kunming Institute of Zoology, Chinese Academy of Sciences, Kunming 650223, China

^2^ Sino-Africa Joint Research Centre, Chinese Academy of Sciences, Kunming, China

^3^ Department of Zoology, Faculty of Life Sciences, University of Ilorin, Ilorin, Kwara State, Nigeria

^4^ Department of Biosciences and Biotechnology, College of Pure and Applied Sciences, Kwara State University, Malete, Kwara State, Nigeria

^5^ Laboratory of Applied Ecology, Faculty of Agronomic Sciences, University of Abomey-Calavi, Benin

^6^ Department of Biological Science, Faculty of Sciences, University of Abuja, Abuja, Nigeria

^7^ Department of Zoology, Faculty of Science, University of Ibadan, Ibadan, Oyo State, Nigeria

^8^ National Park Service Headquarter, Federal Capital Territory, Abuja, Nigeria

^9^ Department of Microbiology, Faculty of Science, University of Ibadan, Ibadan, Oyo State, Nigeria.

^10^ Taraba State Polytechnic, Suntai, Taraba State, Nigeria

^11^ Department of Aquaculture and Fisheries Management, Faculty of Agriculture, University of Ibadan, Ibadan, Oyo State, Nigeria.

^12^ Department of Zoology, Faculty of Science, University of Douala, Douala, Cameroon.

^13^ Department of Zoology, Faculty of Science, University of Dschang, Dschang, Cameroon.

^14^ Wild Forensic Center, Kunming, China

^15^ Department of Biological Sciences, Faculty of Sciences, Federal University Lokoja, Lokoja, Nigeria

^16^ Department of Zoology and Environmental Biology, Faculty of Biological Sciences, University of Nigeria, Nsukka, Nigeria.

^17^ These authors contributed equally to this work

*Correspondence: Lotanna Micah Nneji, lotannanneji@gmail.com; Adeniyi C. Adeola, [chadeola@mail.kiz.ac.cn](mailto:chadeola@mail.kiz.ac.cn); Christopher D. Nwani, [chris.nwani@unn.edu.ng](mailto:chris.nwani@unn.edu.ng)

Table S4: Maximum likelihood partition of the PTP analyses conducted on the bPTP webserver

| Species | Support | Representative Sequence Number for each haplotype (haplotype No) | Matrilineal Group |
| --- | --- | --- | --- |
| Species 1 | 1.00 | DOFKA182 (Hap_8) | Matriline D |
| Species 2 | 0.648 | HVDBF089 (Hap_15), HVDBF569 (Hap_16), HVDBF087 (Hap_14) | Matriline C |
| Species 3 | 0.449 | E15 (Hap_25), H7 (Hap_18), D37 (Hap_22), B40 (Hap_20), Group_3 (Hap_30), H5 (Hap_31) | Matriline E |
| Species 4 | 0.787 | AMNHI490 (Hap_1), DCF653-15 (Hap_19), GBMIN131122-17 (Hap_10), DCF373-15 (Hap_5), GBMIN131026-17 (Hap_9), DCF654-15 (Hap_6) | Matriline G |
| Species 5 | 0.996 | DCF701-15 (Hap_7) | Matriline F |
| Species 6 | 0.961 | HVDB208-10 (Hap_12) | Sub-matriline B (i) |
| Species 7 | 0.326 | BAFEN263-10 (Hap_3), E10 (Hap_23), E4 (Hap_23), BAFEN296-10 (Hap_4), E3 (Hap_27), BAFEN262-10 (Hap_2), D33 (Hap_21) | Matriline A |
| Species 8 | 0.618 | F69 (Hap_29), E13 (Hap_24), E17 (Hap_26) | Matriline A |
| Species 9 | 0.566 | HVDBF242-11 (Hap_16) | Sub-matriline B (ii) |
| Species 10 | 0.490 | HVDB038-09 (Hap_11), HVDBF032-10 (Hap_13) | Sub-matriline B (ii) |

Matrilineal group represents matrilines identified by the phylogenetic analyses
